# The needs and unmet needs for people living with dementia, caregivers and care workers in dementia health care systems: a systematic review

**DOI:** 10.3389/fpubh.2025.1605993

**Published:** 2025-08-20

**Authors:** Michele Sorrentino, Michelangelo Mercogliano, Claudio Fiorilla, Irene Stilo, Federica Esposito, Marcello Moccia, Luigi Lavorgna, Giuseppina Affinito, Elena Salvatore, Maria Pia Sormani, Anna Odone, Azeem Majeed, Fabiana Rubba, Maria Triassi, Raffaele Palladino

**Affiliations:** ^1^Department of Public Health, University “Federico II” of Naples, Naples, Italy; ^2^Department of Public Health, Experimental and Forensic Medicine, University of Pavia, Pavia, Italy; ^3^Department of Molecular Medicine and Medical Biotechnology, Federico II University of Naples, Naples, Italy; ^4^Multiple Sclerosis Unit, Policlinico Federico II University Hospital, Naples, Italy; ^5^Department of Advanced Medical and Surgical Sciences, University of Campania “Luigi Vanvitelli”, Naples, Italy; ^6^Department of Health Sciences, University of Genova, Genova, Italy; ^7^Medical Direction, Fondazione IRCCS Policlinico San Matteo, Pavia, Italy; ^8^Department of Primary Care and Public Health, School of Public Health, Imperial College, London, United Kingdom; ^9^Interdepartmental Research Center in Healthcare Management and Innovation in Healthcare (CIRMIS), Naples, Italy

**Keywords:** dementia, need, systematic review, Alzheimer’s disease, caregiver, healthcare workers, unmet needs

## Abstract

**Introduction:**

The prevalence and costs of dementias are rising due to demographic changes. Dementia care depends largely on informal caregivers and fragmented healthcare systems that often fail to meet the needs of people with dementia.

**Objectives:**

This systematic review aims to identify unmet needs and barriers in European dementia care, providing a framework to improve health strategies.

**Methods:**

Following PRISMA guidelines, articles from 2013 to 2023 were screened from Embase, PsycINFO, HTA Database, and Web of Science. The Mixed Methods Appraisal Tool was used for evaluation.

**Results:**

From 3,738 articles, 47 met the inclusion criteria. Through a narrative synthesis, the review identified unmet needs and barriers among People Living with Dementia, caregivers, and healthcare workers. Psychosocial and emotional support are essential for managing stress and ensuring quality of life. Caregivers demand education about dementia care, progression, and self-care, while healthcare workers need training, and interdisciplinary teams. Cultural sensitivity is critical for addressing stigma and facilitating inclusive care for ethnic minorities. Healthcare access remains fragmented, thereby decreasing continuity of care for families. High costs, bureaucratic complexity, and geographical inequalities, particularly in rural areas can be barrier to care for People Living with Dementia and their families. The COVID-19 pandemic disrupted social support services, increasing distress and uncertainty. About limitation, publication bias and geographical bias from focus on Europe were possible, potentially overlooking insights from other regions.

**Conclusion:**

There is need for public policies to enhance education, community support, and dementia awareness, with a focus on culturally sensitive care.

## 1 Introduction

Alzheimer’s disease (AD) and other dementia constitute a complex set of progressive neurodegenerative conditions that primarily affect older adults (1). Both are recognized as leading causes of disability in the older adult (2). In 2019, approximately 14.1 million people were living with AD or other forms of dementia in Europe alone, a number projected to double by 2050 (3).

Median life expectancy is around 3 to 6 years after formal diagnosis of dementia but some individuals survive for as long as 20 years (4). Clinical deterioration is progressive and ranges from mild or early stage of dementia (e.g., forgetful, some language difficulties, and mood changes) for the first year or two, the moderate or middle stage (e.g., very forgetful, increasing difficulty with speech, and help needed with self-care activities) from the second to the fourth or fifth years, and the severe or late stage (e.g., serious memory disturbances and nearly total dependence and inactivity) from the fifth year onwards (5). Disability progression and increase in seeking-care lead to a significant drop in overall quality of life (6).

While most care needs for people living with dementia (PLWD) are satisfied by their caregivers (someone who takes care of a person, usually a family member) (6), this might have a negative impact on carer’s physical and mental wellbeing and also their social life and financial situation (7). Often, caregivers often experience elevated levels of stress and depression, and reduced employment compared to the general population (8–10). However, several factors, including resilience, post-traumatic growth, and a positive attitude, influence the disease burden (11).

Healthcare workers (HCWs) play a crucial role in delivering care to patients with advanced-stage dementia, where professional support becomes indispensable due to the progression of cognitive impairment and frailty (12). Nevertheless, this responsibility often places HCWs under considerable pressure, leading to both psychological and physical strain, including an increased risk of injury and depression (13). As a result, HCWs face a heightened higher risk of stress (14) and burnout (15), further compounding the challenges they encounter in their demanding roles. Furthermore, high staff turnover rate (16) and training programs held by more experienced professionals rather than qualified instructors (17) represent a barrier in delivering care in a highly complex setting.

Considering the complexity of dementia care and the challenges faced by all those involved, it is essential to identify and understand the specific needs encountered at different levels of the care system. The central question guiding this review is as follows: What are the needs, unmet needs, and linked barriers experienced by primary, secondary, and tertiary end-users (people living with dementia, caregivers, healthcare workers, and other stakeholders) within dementia care systems? Answering this question can provide valuable insight into the challenges encountered at different levels of engagement with dementia-related services, with the ultimate aim of informing targeted interventions and contributing to improvements in the overall care and support ecosystem.

## 2 Methods

This systematic review analysed studies focusing on needs, unmet needs and barriers in European dementia care-systems and was conducted using the Preferred Reporting Items for Systematic Reviews and Meta-Analyses (PRISMA) guidelines (18). Refer to the Supplementary Table 1 for additional details.

### 2.1 Search strategy and eligibility criteria

PubMed was used as a primary dataset. Additional database searches were performed in the following databases: Embase, PsycINFO (EBSCOhost), Health Technology Assessment Database, and Web of Science (Clarivate). Duplicate were eliminated using Rayyan AI (19). These searches covered 10 years (2013–2023). Eligibility criteria are summarized in Table 1.

**Table 1:** Eligibility criteria.

Domains	Eligibility Criteria
**Population (P)**	People living with dementia or Alzheimer’s Disease with a formal diagnosis Caregivers of people living with dementia Healthcare workers and other stakeholders involved in dementia care
**Intervention/Outcome (I/O)**	Assessment or search of: Needs expressed Unmet needs Barriers to fulfilling dementia care needs
**Geographical Area (S)**	European setting
**Timeframe (T)**	From 01/01/2013 to 31/12/2023
**Other criterial**	Written in English Original Research Studies published in or after 2013

The search strategy employed a combination of keywords and Boolean operators (Table 2). The keywords used to identify the needs, the unmet needs, and the barriers that compromise the assistance of people living with dementia, their caregivers, and HCWs. The keywords, aligned the PICO framework, include the following terms: Population (P) (“Alzheimer Disease” OR “Dementia”) AND Intervention/Outcome (“Barrier*” OR “Need*” OR “Access” OR “Healthcare” OR “Health Care Utilization”) AND Geographical Area (S) (“Europ*” [MeSH]) AND Timeframe (T) (“2013/01/01” [PDAT]: “2023/12/31” [PDAT]). No comparison was made.

**Table 2:** Research string divided by domains.

Domains	Keywords
**Study Population (P)**	"Alzheimer Disease" OR "Dementia"
AND	
**Intervention/Outcome (I/O)**	“Barrier*” OR “Need*” OR “Access” OR “Healthcare” OR “Health Care Utilization”
AND	
**Comparison (C)**	Not Applicable
AND	
**Geographical Area (S)**	"Europ*" [MeSH]
AND	
**Timeframe (T)**	"2013/01/01"[PDAT]: "2023/12/31"[PDAT]

### 2.2 Data extraction and quality assessment

Five reviewers (MS, MM, CF, IS, FE) examined titles and abstract to identify studies adhering to inclusion criteria. If the abstract lacked sufficient information to decide for inclusion or exclusion, full text review was performed. Conflict and uncertainties were discussed with the senior reviewer (RP). Articles selected trough title-abstract analysis were fully review. During full text review, additional data was extracted and summarized in Excel spreadsheets. The quality of the selected papers was assessed using the revised version of the Mixed Methods Appraisal Tool (MMAT) (20). This tool is designed to evaluate different dimensions of study quality according to the specific research design. Studies were not automatically excluded due to quality concerns; however, those with lower quality were closely examined to understand their potential influence on the overall findings. Each paper was independently reviewed by the evaluators to ensure an unbiased assessment of its quality.

## 3 Results

### 3.1 Study selection

The main search identified 3,854 studies. After adapting and running the research string on secondary database, duplicates were removed using Rayyan AI,^1^ and a database of 3,175 unique studies was compiled and 40 studies were identified through reference list. Upon review of their titles and abstracts, 3,038 studies were deemed irrelevant and excluded. Subsequently, 177 publications underwent a full-text review, resulting in the selection of 47 studies meeting the inclusion criteria. Grey literature was not considered, as well as conference papers, dissertations, letters, and editorials.

The 130 studies were excluded for the following reasons: 92 lacked assessments of needs or unmet needs, 25 were excluded due to study design/type, 8 were not set in a European nation, 3 were not focused on the population with Alzheimer’s disease and other dementias, and 2 studies were excluded due to lack of full-text availability. The list of excluded articles and the detailed reasons for their exclusion are provided in the Supplementary Table 2.

**Figure 1:**
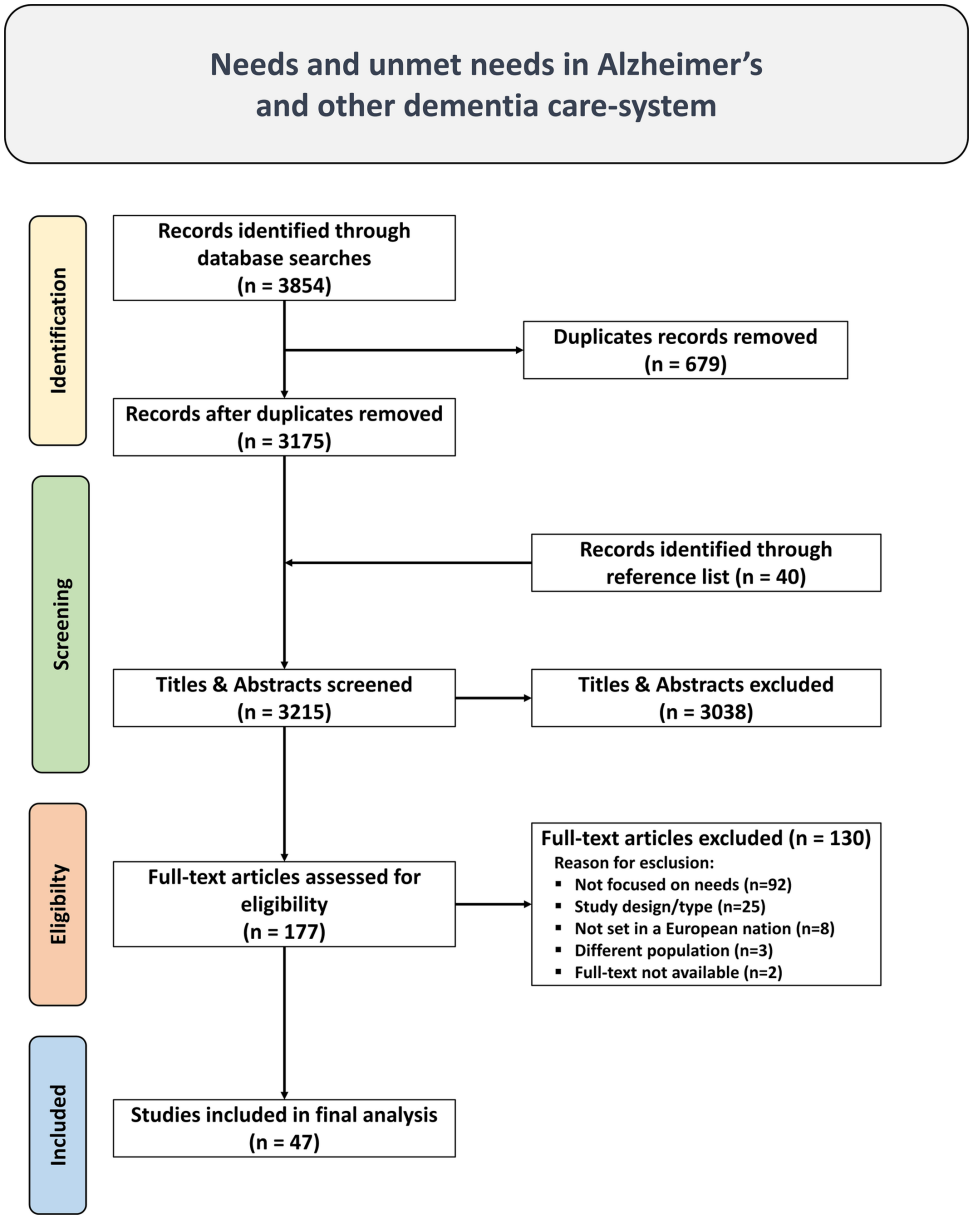
PRISMA Flow diagram of literature search, abstract screen, full article assessment for exclusion and inclusion criteria with most common reasons for exclusion detailed.

A visual representation of this selection process and the reason for exclusion is provided in the PRISMA diagram (Figure 1).

### 3.2 Study characteristics

The characteristics of the articles included are summarized in Table 3. 47 studies, spanning from 2013 to 2023, were selected for this review. The publication years were: five studies in 2013 (21–25), five studies in 2014 (26–30), one in 2016 (31), two in 2017 (32, 33), five in 2018 (34–38), five in 2019 (39–43), eleven in 2020 (44–54), nine in 2021 (55–63), three in 2022 (64–66), and one in 2023 (67). From the COVID-19 pandemic event, some studies were conducted during the pandemic with varying degrees of focus on COVID-19: three studies explicitly focused on COVID-19 (47, 55, 58), one studies did not mention COVID-19 despite being conducted during the pandemic (64) and three studies (62, 65, 66) combined pre-pandemic data with observations during the pandemic without focusing on COVID-19.

**Table 3:** Study characteristics, results, and key finding.

(A)								
Ref ID	Study characteristics							
	Authors	Year	COVID-19	Study design	Study methodology	Nation of study	Population	Key settings discussed
(40)	Abreu W et al.	2019	Pre-pandemic	Cross sectional study	Survey	Portugal	83 PLWD	Home-based care
(25)	Bakker C et al.	2013	Pre-pandemic	Cross sectional study	Survey	Netherlands	215 PLWD and their caregiver	Clinical or hospital-based care
(26)	Bakker C et al.	2014	Pre-pandemic	Community-based prospective study	Survey	Netherlands	215 PLWD and their caregiver	Community-based care
(56)	Barry HE et al.	2021	Pre-pandemic	Qualitative study	Interview	United Kingdom	33 (15 Caregiver; 18 PLWD)	Home-based care
(37)	Saloua Berdai Chaouni et al.	2018	Pre-pandemic	Qualitative study	Interview	Belgium	25 (13 HcP; 12 Caregiver) Minority group: Moroccan	Home-based care, community-based care, and clinical or hospital-based care
(27)	Bökberg C et al.	2014	Pre-pandemic	Qualitative study	Focus Group/Interview	Sweden	23 HcP	Clinical or hospital-based care, home-based care, residential or long-term care facilities
(34)	Bökberg C et al.	2018	Pre-pandemic	Cross sectional study	Survey	Sweden	177 PLWD	Home-based care
(58)	Carcavilla N et al.	2021	During pandemic, focused on Covid-19	Cross sectional study	Survey	Spain	106 Caregiver	Community-based care
(32)	De Cola MC et al.	2017	Pre-pandemic	Qualitative study	Survey/Interview	Italy	59 Caregiver	Home-based care
(64)	Dibao-Dina C et al.	2022	Pre-pandemic and during pandemic but COVID-19 is not mentioned	Cross sectional study	Mixed method – survey	16 European countries (Intermediate care for dementia in Europe): Bosnia, Croatia, Georgia, Greece, France, Hungary, Ireland, Israel, Italy, Latvia, Poland, Portugal, Romania, Switzerland, the United Kingdom, Ukraine	583 HcP	Primary care, home-based care, community-based care, residential or long-term care facilities.
(21)	Dickinson C et al.	2013	Pre-pandemic	Qualitative study	Interview	United Kingdom	46 (29 Caregiver; 17 PLWD)	Home-based care
(31)	Eichler T et al.	2016	Pre-pandemic	Cross sectional study	From data of a randomized controlled trial	Germany	227 PLWD	Primary care
(59)	Farina N et al.	2021	Pre-pandemic	Qualitative study	Interview	United Kingdom	30 (15 dyads)	Home-based care
(45)	Foley T et al.	2020	Pre-pandemic	Qualitative study	Focus group	Ireland	32 HcP	Community-based care and clinical or hospital-based care
(46)	Frias CE et al.	2020	Pre-pandemic	Cross sectional study	Survey	Spain	160 Caregiver	Home-based care, primary care
(57)	Froelich L et al.	2021	Pre-pandemic	Longitudinal cohort study	Survey	Germany, Spain, the United Kingdom	1,232 (616 dyads)	Community-based care
(47)	Giebel C et al.	2020	During pandemic, focused on Covid-19	Qualitative study	Interview	United Kingdom	50 (42 Caregiver; 8 PLWD)	Home-based care
(33)	Gove D et al.	2017	Pre-pandemic	Qualitative study	Focus group/Interview	United Kingdom	23 HcP	Primary care
(48)	Hossain MZ et al.	2020	Pre-pandemic	Qualitative study	Focus group/Interview	United Kingdom	27 Caregiver Minority group: Bangladeshi	Home-based care and community-based care
(38)	Janssen N et al.	2018	Pre-pandemic	Longitudinal cohort study	Survey	8 European countries (Actifcare project): the Netherlands, Germany, the United Kingdom, Ireland, Sweden, Norway, Portugal, Italy	451 Caregiver or PLWD	Community-based care
(50)	Janssen N et al.	2020	Pre-pandemic	Cross sectional study	Survey	8 European countries (Actifcare project): the Netherlands, Germany, the United Kingdom, Ireland, Sweden, Norway, Portugal, Italy	896 (447 dyads)	Community-based care
(35)	Kerpershoek L et al.	2018	Pre-pandemic	Cohort study	Survey	8 European countries (Actifcare project): the Netherlands, Germany, the United Kingdom, Ireland, Sweden, Norway, Portugal, Italy	902 (451 dyads)	Home-based care
(65)	Leroi I et al.	2022	Pre-pandemic and during pandemic but COVID-19 is not mentioned	Cross Sectional study	Mixed Method - Survey/Focus group	United Kingdom, France, Cyprus	194 (97 Caregiver; 97 PLWD)	Community-based care
(29)	Malthouse R et al.	2014	Pre-pandemic	Qualitative study	Interview	United Kingdom	10 (5 Caregiver; 5 PLWD)	Clinical or hospital-based care
(67)	Mank A et al.	2023	During pandemic but COVID-19 is not mentioned	Cross sectional study	Survey	Netherlands	270 Caregiver	Home-based care, clinical or hospital-based care
(41)	Mazurek J et al.	2019	Pre-pandemic	Cross sectional study	Survey	Poland	88 (41 Caregiver; 47 PLWD)	Home-based care, community-based care and primary care
(66)	Michelet M et al.	2022	Pre-pandemic and during pandemic but COVID-19 is not mentioned.	Longitudinal study	Survey	8 European countries (Actifcare project): the Netherlands, Germany, the United Kingdom, Ireland, Sweden, Norway, Portugal, Italy	451 PLWD	Home-based care
(44)	Minaya-Freire A et al.	2020	Pre-pandemic	Qualitative study	Survey/Focus group	Spain	10 HcP	Clinical or hospital-based care
(22)	Claudia Miranda-Castillo et al.	2013	Pre-pandemic	Qualitative cross sectional study	Survey/Interview	United Kingdom	280 (128 Caregiver; 152 PLWD)	Home-based care
(49)	Mitchell G et al.	2020	Pre-pandemic	Qualitative study	Focus group/Interview	United Kingdom	20 PLWD	Community-based care
(51)	Monsees J et al.	2020	Pre-pandemic	Qualitative study	Interview	Germany	8 Caregiver Minority group: Turkish	Home-based care
(42)	Moreno-Cámara S et al.	2019	Pre-pandemic	Qualitative study	Focus group	Spain	82 Caregiver	Home-based care, community-based care and clinical or hospital-based care.
(60)	Nielsen TR et al.	2021	Pre-pandemic	Qualitative study	Focus group/Interview	Denmark	35 (23 HcP; 12 Caregiver) Minority group: Turkish, Pakistani, and Arabic-speaking minority ethnic groups.	Home-based care and community-based care
(39)	Oliveira D et al.	2019	Pre-pandemic	Qualitative study	Mixed method - Focus group/Interview	United Kingdom	73 (46 HcP; 27 Caregiver)	Community-based care and home-based care
(23)	Page S et al.	2013	Pre-pandemic	Cross sectional study	Survey	United Kingdom	40 HcP	Community-based care, clinical or hospital-based care, research and special settings
(36)	Pini S et al.	2018	Pre-pandemic	Qualitative study	Interview	United Kingdom	42 Caregiver	Community-based care
(43)	Quinn C et al.	2019	Pre-pandemic	Cohort study	Survey	United Kingdom	1,283 Caregiver	Community-based care
(55)	Rusowicz J et al.	2021	During pandemic, focused on Covid-19	Cross sectional study	Survey	Poland	85 Caregiver	Home-based care
(61)	Ryan L et al.	2021	Pre-pandemic	Qualitative study	Interview	Ireland	34 (14 HcP; 20 Caregiver)	Community-based care
(62)	Schnelli A et al.	2021	Pre-pandemic and during pandemic but COVID-19 is not mentioned	Qualitative study	Interview	Switzerland	5 PLWD	Home-based care, clinical or hospital-based care
(28)	Felicity Smith et al.	2014	Pre-pandemic	Qualitative study	Interview	United Kingdom	19 (14 Caregiver; 5 PLWD)	Home-based care
(24)	Somme D et al.	2013	Pre-pandemic	Qualitative study	Survey/Interview	France	974 HcP	Primary care
(52)	Telenius EW et al.	2020	Pre-pandemic	Qualitative study	Interview	Norway	35 PLWD	Home-based care
(63)	Timmons S et al.	2021	Pre-pandemic	Cross sectional study	Survey	Ireland	69 HcP	Residential or long-term care facilities
(30)	Nienke van Wezel et al.	2014	Pre-pandemic	Qualitative study	Focus group/Interview	Netherlands	28 Caregiver Minority group: Turkish, Moroccan, and Surinamese Creole family carers	Home-based care and community-based care
(53)	Varik M et al.	2020	Pre-pandemic	Qualitative study	Interview	Estonia	16 Caregiver	Home-based care, residential or long-term care facilities
(54)	Vullings I et al.	2020	Pre-pandemic	Qualitative study	Interview	Netherlands	20 (1 HcP; 14 Caregiver; 5 PLWD)	Home-based care

(B)							
Ref ID	Results and key findings						MMAT
	Psychosocial and emotional needs	Educational and informational needs	Cultural needs	Healthcare needs	Barriers to care	Key findings	
(40)	Need for support for managing functional dependency and frailty.			Both PLWD and their caregivers expressed significant physical and environmental needs, such as medication management, fall prevention, sensory support, food preparation, and money management.		A high number of participants were classified as “severe dementia”, “fully dependent”, “severely or fully dependent in the activities of daily living” and/or “severe frailty.” Needs increased with the progression of symptoms and severity. The main areas of need were: food preparation, medication, toilet use, interaction, eating and drinking, memory, sleeping, and falls prevention. These needs were present in both severe dementia and frailty. Needs persisted independently of home-care programs. There is a need for more tailored interventions that also address the emotional and psychological needs of caregivers and PLWD.	100%
(25)	Caregiver express necessity for psychological, emotional, and social support to manage the stress burden associated with caregiving.					Caregivers of patients with more unmet needs reported lower vitality, poorer general health, more difficulties in daily tasks due to physical problems, and increased physical pain. Unmet needs were linked to greater limitations in social activities and higher mental health issues. Caregivers lacking psychological support, like psychosocial assistance or respite care, were particularly at risk of social limitations and feelings of depression and anxiety.	100%
(26)	Psychological support to manage neuropsychiatric symptoms associated with unmet needs increasing during the progression of dementia.				Informal caregivers face barrier in accessing support for social, intimate, and informational needs.	PLWD experienced high levels of unmet needs in areas such as daytime activities, social company, intimate relationships, and information. Unmet needs were associated with an increase in neuropsychiatric symptoms. Contrary to their caregivers, PLWD reported a significant decrease in the number of areas with unmet needs over time. Patients also reported fewer unmet needs than caregivers.	100%
(56)	Necessity for psychological, emotional, and social support to manage the stress burden associated with caregiving and reducing anxiety about medication management.	Need for specific information about the disease and the available support, including education on medication management and how to address related anxiety.				PLWD rely on their carers for medicines management, and carers play a significant role in ensuring adherence. Both groups trust healthcare professionals and value the assistance of community pharmacies. PwD have strong beliefs in the necessity of their medicines but have lower levels of concern about their medication, overuse, and potential harm.	100%
(37)			Need for culture-sensitive tests and language support in dementia care.			Results indicate a specific experience of dementia among the Moroccan population, influenced by accumulated “invisibilities” throughout the dementia progression and the need for culture-sensitive tests and language support in dementia care.	100%
(27)		HCWs have numerous educational needs in dementia care, including training in dementia diagnosis, behavior management, education on dementia management, assessment, interventions, and national dementia strategies.				Main findings: Adapting care and services to the individual needs of the person with dementia and their next of kin, along with providing proactive care throughout the disease trajectory, is considered best practice.	100%
(34)	PLWD expressed a high need for psychological support for activities of daily living and psychological distress.			People with severe dementia have a greater need for informal care for activities of daily living, and the need to utilize formal care services increases with the severity of dementia.	Barriers to intermediate care for dementia include high costs, disorientation, exacerbation of behavioral and psychological symptoms, and feelings of shame and guilt.	Key findings include the significant use of informal care by individuals with severe dementia for ADL assistance and supervision, and higher utilization of formal care services by those with the most severe cognitive impairments.	100%
(58)	During the COVID-19 pandemic, caregivers expressed an increased need for psychological support to manage high stress levels due to isolation and the new challenges arising from emerging social and health difficulties.					Caregivers experienced psychological issues such as anxiety, mood disturbances, sleep disorders, and eating problems during the confinement due to COVID-19. They also felt a lack of support when managing challenging behaviors or providing meaningful activities for their patients.	80%
(32)	Necessity for psychological, emotional, and social support to manage the stress burden associated with caregiving.	Educational need for non-pharmacological management of dementia and comprehensive information on disease management.		Family caregivers expressed a need for both instrumental and formal support from HCWs to expedite diagnosis and ensure effective treatment.		Caregivers experienced stress related to dementia. They were not capable of emotional management regarding anxiety, anger, and frustration; furthermore, bivariate analysis highlighted relationship problems and social isolation. Difficulty in managing anger was associated with a decrease in the time spent on their own needs. 47.27% of caregivers reported a sense of guilt. Caregivers believe the services offered by the local government are inadequate.	100%
(64)		Need for continuing education and training, pointing out a lack of information and regular updates on the management of intermediate care services in their areas.	Need to reduce the stigmatization families face when seeking intermediate care.		Barriers to intermediate care include high costs (and financial support), disorientation, exacerbation of behavioral and psychological symptoms, living in rural areas, and feelings of shame, sadness, and guilt.	For intermediate care, the main difficulties are the high cost, family shame, disorientation, and behavioral issues caused by moving to a new environment. Across Europe, there is inconsistency in care availability. General Practitioners highlight concerns about costs and complex administrative procedures. Benefits of intermediate care include better medical care, improved quality of life for caregivers, prevention of caregiver burden, and a break for caregivers.	100%
(21)					Need of support to navigate advanced care planning and reducing constraints on choices. Difficulties finding the right time for discussions. Preference for informal plans.	Lack of knowledge and awareness, difficulty in finding the right time, a preference for informal plans over written documentation, constraints on choice around future care, and lack of support to make choices about future healthcare.	100%
(31)	Support to manage the stress burden associated with caregiving.	Need for specific information about the disease and the available support, including social counseling and legal assistance.		Needs of nursing care and treatment, drug treatment and care, medical diagnosis and treatment, special therapies, social counseling, and legal support.		A total of 1,991 unmet needs were identified; the only factor that was significantly associated with the number of unmet needs was higher impairment in ADLs: the number of needs increases with the decline of the functional status.	100%
(59)				Both PLWD and their caregivers expressed significant needs for support for physical activity.	Lack of time and their caregiving role are significant barriers to physical activity.	Physical health is a common barrier for both PLWD and caregivers in maintaining their emotional and physical wellbeing. While caregivers support the physical activity of PLWD, they often have limited time for their own physical activity.	100%
(45)		Physiotherapists need further training in dementia care and evidence-based physiotherapy guidelines to better understand their role.				Physiotherapists reported a significant dementia-related workload. Challenges included lack of formal diagnosis, clinical uncertainty, limited resources, physical environment, and assessing rehabilitation potential. Participants wanted more dementia training and evidence-based physiotherapy guidelines, focusing on communication techniques, cognitive screening tools, dementia sub-types, and ethical issues.	100%
(46)	Necessity for psychological and social support to manage the stress burden associated with caregiving.					Male caregivers reported slightly better quality of life than female caregivers. Significant correlations between caregivers’ quality of life and caregiver burden, psychological wellbeing, and negative aspects of caregiving on health.	80%
(57)	As the disease progressed, the burden on caregivers gradually increased, necessitating enhanced emotional support.			Needs increase with the severity of dementia, as well as the need for access to healthcare resources.		The progression of Alzheimer’s Disease leads to a significant increase in caregiver burden, as well as a decline in the health-related quality of life for both patients and caregivers. Compared to PLWD, caregiver self-reported decline of quality of life, suggesting a discrepancy in the perceived quality of life between PLWD and their caregivers.	100%
(47)	During the COVID-19 pandemic, both caregivers and patients expressed an increased need for support to manage the impact of service disruptions, high stress levels due to isolation and the new challenges arising from emerging social and health difficulties.				During the pandemic, access to healthcare and medications became more difficult, with new barriers arising due to the pandemic. Families were isolated without assistance, increasing concerns about the resumption of care provision after the pandemic.	Thematic analysis identified three overarching themes during pandemic: Loss of control; Uncertainty; Adapting and having to adapt to the new normal. Carers and PLWD were greatly affected by the sudden removal of social support services, and concerned about when services would re-open. Carers were worried about whether the person they cared for would still be able to re-join social support services.	100%
(33)			GPs expressed a need for cultural competence in society and healthcare to address stigma and improve societal perceptions of dementia and to improve their own communication with individuals with dementia.			Most GPs perceive people with dementia as failing to reciprocate, linked to societal stigma, affecting timely diagnosis and quality of care.	100%
(48)			Caregivers highlighted the need for a deeper understanding of cultural and religious values to improve cultural competence.			In the United Kingdom, Bangladeshi caregivers highlighted the need for a deeper understanding of cultural and religious values to improve cultural competence and reduce barrier to access healthcare services.	100%
(38)	Negative relationship observed between these unmet needs and lack of time with quality of life.				Lack of time and their caregiving role are significant barriers to right timely care. Another barrier are costs associated with care.	Unmet needs significantly lower the quality of life for people with dementia and their time to care.	100%
(50)	PLWD expressed a high need for psychological support due to the significant stress associated with unmet social needs.	Caregivers demonstrated a significant need for information about healthcare services and the management of dementia symptoms.		HCWs and PLWD emphasized the need for a proactive approach to person-cantered care (24, 36).		Four need profiles in PLWD were identified. “No Need Profile” (41% of the sample): no unmet care needs or need for immediate interventions. “Met Psychological Needs Profile” (25%): psychological needs are adequately addressed for both caregivers and PLWD. “Unmet Social Needs Profile” (15%): unmet social needs that increase caregiver stress. “Met Social Needs Profile” (19%): addressing social needs improves quality of life. Increasing unmet needs can lead to higher stress and reduced quality of life for both caregivers and PLWD.	100%
(35)	PLWDs rated their unmet social needs significantly lower than their caregivers, with a negative relationship observed between these unmet needs and both their own and their caregivers’ quality of life.					Informal carers reported almost twice as many needs as people with dementia. These differences in perspective may lead to conflicts in decision-making regarding accepting care. Professionals should pay more attention to and be trained in dealing with these decisional conflicts.	100%
(65)	PLWD with hearing and/or visual impairment also expressed the need for psychological support.	PLWD with hearing and/or visual impairments need more education on the use of assistive devices.		PLWD with hearing or visual impairments need integration of services to address the increasing difficulties. Both PLWD and their caregivers expressed significant physical and environmental needs, such as sensory support, fall prevention, and support for physical activity.		Over 94% of participants reported having unmet support care needs, with a median of 13 items indicating some level of need. This wide range of unmet needs across psychological, physical, and informational domains suggests a complex interaction between sensory impairments, unmet support care needs, and quality of life, necessitating a tailored approach to care.	100%
(29)					PLWD face barriers to physical activity due to physical health and cognitive impairment, with dementia progression increasing the need for physical activity while activity levels decrease. Caregivers face barriers in improving their quality of life due to fear of missing out, safety concerns, and social challenges related to physical activities.	General health benefits of physical activity are less investigated in Alzheimer’s disease. PLWD fear getting lost and safety concerns, limiting activity opportunities. Previously enjoyed activities are more likely to continue with adjustments. Social interactions are valued but hindered by misunderstandings of dementia. Dementia progression shifts couple dynamics, affecting activity levels. Established routines support activity but are vulnerable to changes in health and mood.	100%
(67)	The need for support and assistance in managing the behavioral and neuropsychiatric symptoms of the PLWD, reducing distress and depressive symptoms, and improving quality of life.					Behavioral symptoms and functional impairment in patients correlate with more informal care time, caregiver distress, depressive symptoms, and lower quality of life in care partners.	80%
(41)	PLWD expressed a high need for psychological support due to the significant stress associated with daily living, psychological distress and the need for companionship.					The most commonly reported unmet needs by both people with dementia and their carers encompassed activities of daily living, psychological distress, and the need for companionship, with carers identifying significantly more needs than the individuals with dementia. Neither group highlighted unmet needs in areas related to caregiving for others or abuse/neglect.	100%
(66)	PLWD have negative relationship between unmet needs and the level of neuropsychiatric symptoms, caregivers demand support for their emotional and psychological well-being.					Unmet needs for daytime activities and for company were associated with more affective symptoms at baseline, six and 12 months, and with more psychotic symptoms at baseline.	100%
(44)		Need for educational programs related to dementia care (e.g., pain management), to have better communication and collaboration within the team, more time and less work overload.				The main barriers they identified were lack of knowledge and skills and lack of time. The participants proposed two main improvements: (1) a training program consisting of three courses (pain evaluation and management, dementia and pain, and pharmacology) and (2) the creation of a specific register for nurses to record patients’ pain.	100%
(22)	Necessity for psychological, emotional, and social support to manage the stress burden and anxiety associated with caregiving.	Need of information about the disease and the available support, including social counseling.				PLWD felt capable of managing their medications, but caregivers played a crucial role in ensuring adherence. They trust in healthcare professionals but highlighted a need for more information. With dementia progression, the role of caregivers increased.	100%
(49)			Need to encourages reconsideration of public perceptions and acknowledgment of the capabilities rather than disabilities of individuals with dementia. This public perceptions impacting on PLWD well-being.			People living with dementia often experience discrimination by members of society who believe they are no longer capable of managing their everyday lives adjust focus on their disabilities. Public misconceptions about dementia still prevalent despite improvements and reduce PLWD well-being.	100%
(51)		Necessity for specific information about the disease and the available support, including social counseling and legal assistance.	There is a need for better access to relevant information and the inclusion of minority cultures in healthcare services. Key needs include access to culturally sensitive services, caregiver support for managing dementia within cultural contexts, cultural competence training, engagement with communities.		Need for financial support to cope with the high costs of intermediate care for dementia. Cultural barriers in accessing care and need to have a culturally sensitive healthcare services.	There is an inclination within Turkish families to provide care without considering healthcare due to limited access to culturally sensitive services, even if they have limited prior knowledge of dementia. Some challenges include aggressiveness and night-time activity of the person with dementia, as well as the physical and mental strain on caregivers.	100%
(42)	Necessity for psychological, emotional, and social support.	Need education in managing various aspects of dementia, particularly at the beginning of the care process and throughout the care pathway, including special care situations and behavioral problems.				Family caregivers’ needs are complex and interrelated. Interventions addressing multiple care needs can improve their emotional health. Psycho-educational support for managing dementia and caregiver well-being is essential, and involving the whole family in these interventions is beneficial. Proactive strategies should focus on enhancing negotiation, planning, and accessing external resources, with new technologies aiding in-home caregiving.	100%
(60)			Minority groups require increased cultural sensitivity and competence in healthcare interactions, as well as improved awareness and understanding of dementia within their communities.			In Denmark, HCWs and caregivers from Turkish, Pakistani, and Arabic-speaking minority groups report difficulties due to a lack of knowledge regarding available services, misconceptions and stigma related to dementia within their own communities, a lack of services that focus on social needs, and a complex healthcare system that is difficult to navigate.	100%
(39)	Caregivers expressed the need for support respite breaks.	Caregivers expressed the need for information, awareness, and education on self-care health behaviors.		Family caregivers expressed a strong need for financial support.		Several service- and individual-related factors may affect health-promoting self-care behavior in family carers. Service-related factors represent service organization and coordination, staff attitudes, and need for more respite care, continuity of care and support, and education and awareness. Individual-related factors represent carer difficulties to prioritize their own needs, the health impact on the ability to self-care, relationships and feelings, coping strategies, and financial strain.	100%
(23)	Necessity for psychological, emotional, and social support to manage the stress burden associated with caregiving.	Need of education on dementia management, assessment, creating dementia-friendly environments. Interventions, and national dementia strategies. HCP expressed the need for educational programs related to dementia care, and to have better communication and collaboration within the team.				Nurses had varying levels of confidence in dementia care. They felt competent in dementia assessment and interventions, with some gaps in psychosocial aspects like communication and person-centered care. Nurses reported gaps in biomedical knowledge, especially in pharmacological approaches and managing depression. The most significant gap was identified in legal and ethical aspects. Nurses recognized the need for further specialized training to address their gaps and improve dementia care delivery.	100%
(36)	Necessity of social support to enhance relationships and to manage the stress burden associated with caregiving.				Barriers in organizing support and care, often involving multiple agencies, professionals, friends, and family members, and find that bureaucracy adds frustration and is time-consuming.	Caring for their relative with dementia restricted and eroded the carers’ sense of being able to fulfill their needs; the ability to feel in control of day-to-day life and the future. They feel needs to have time and space for relaxation or spontaneity; and their need to feel connected to others.	100%
(43)	Necessity for emotional and psychological support to manage the stress burden associated with caregiving.					Lower well-being was associated with low caregiving competence, perceiving fewer positive aspects of caregiving, high caregiving stress, and high role captivity. Lower satisfaction with life was associated with low caregiving competence, perceiving fewer positive aspects of caregiving, high caregiving stress, and high role captivity.	100%
(55)	Increased need for psychological support to manage high stress levels due to isolation.				During the pandemic, access to healthcare and medications became more difficult, with new barriers arising due to the pandemic. Families were isolated without organizational support or assistance with difficult in managing daily errands and protective measures during the pandemic.	Caregivers experienced high levels of stress, with significant health deterioration reported for both caregivers and their charges. Over 83% received no offer of help in key areas during the pandemic.	100%
(61)				HCWs and PLWD emphasized the need for a proactive approach to person-cantered care to improve access to community based services.	HCWs and caregivers recognized emotional barriers to community care access due to reluctance to question authority and fear of stigma, expressing the need to improve access to community-based services for caregivers and user-cantered models of care to facilitate better access and improve the quality of care.	Key finding was the significant impact of internal emotional barriers on the utilization of CBS. Despite recognizing external expressions of emotional discomfort, PHPs had limited awareness of the deeper, internal emotional barriers, indicating a gap in understanding and support for caregivers within the care pathway.	100%
(62)		Demanding online resources that focus on the course of the disease and related issues.				Were identified specific needs of persons with dementia regarding information on the internet. Information should focus on the course of disease and related issues.	100%
(28)		Necessity for specific information about the disease and the available support, advices and education on medication management.				Caregivers reported difficulties in maintaining supplies, ensuring adherence to regimens and accessing health professionals. Caregivers reported difficulty in find advice and information about medicines.	100%
(24)		Healthcare professionals emphasize the importance of non-pharmacological management of dementia, effective communication with families and PLWD.				Behavioral disorders represented the most common problem encountered, while half of the GPs considered management of comorbidities easy roles to fulfill. GPs finding coordination of care easy. Half of the GPs interviewed considered themselves insufficiently trained on non-drug treatments.	100%
(52)	Need for psychological and social support to manage the stress burden associated with caregiving					“Need to be who I am”: to stay connected; to be active and participate; and to live for the moment.	100%
(63)		Nurses require training in palliative care, pain management, and medication management.				Staff were confident in their ability to implement change but were de-motivated. Staffing levels, managing risk during change, and perceived reluctance in others were common barriers. Pain assessment and management were the most identified learning needs.	80%
(30)			Communities need support in managing the emotional and social challenges of caregiving in culturally diverse contexts.			Communities need support in managing the emotional and social challenges of caregiving in culturally diverse contexts. Highlights the negative aspects and burdens of caregiving, including loneliness and witnessing deterioration.	100%
(53)	Unmet social needs for psychological, emotional, and social support.	Caregivers expressed a strong desire for training and groups to facilitate information sharing (e.g., information on technological devices).	Caregivers expressed the need for more culturally informed care to enhance dementia care.	Need of organization and coordination of services to ensuring continuity of support, that is essential to promoting self-care health behaviors among PLWD	Informal caregivers express the need to access support services.	Awareness and support services help to alleviate the negative effects of caregiving on carers. Carers felt a major responsibility and sometimes experienced a sense of hopelessness due to insufficient support. There is a lack of appropriate information and professional advice on how to access support and care for their relatives.	100%
(54)	Caregivers expressed the need for support groups for emotional well-being.	They require information on dementia progression, disease management, and available care and support options.			Barriers to dementia care are the need for easier access to care due to the complexity and fragmentation of the care system, improve in case management and information provision.	Participants underline importance of social networks in assisting daily tasks, the lack of time for non-medical activities in formal care, information, emotional support by considering the variation of satisfaction with case management, and easier access to care. Participants expressed a need for more information, emotional support, and simpler access to care services, considering complexity and financial challenges	100%

Most of the studies were conducted in single nations. The United Kingdom had the highest number of studies (*n* = 14) (21–23, 28, 29, 33, 36, 39, 43, 47–49, 56, 59), followed by the Netherlands (*n* = 5) (25, 26, 30, 54, 67), Spain (*n* = 4) (42, 44, 46, 58), Ireland (*n* = 3) (45, 61, 63), Germany (*n* = 2) (31, 51), Sweden (*n* = 2) (27, 34), Poland (*n* = 2) (41, 55), Portugal (*n* = 1) (40), Norway (*n* = 1) (52), Belgium (*n* = 1) (37), Estonia (*n* = 1) (53), Denmark (*n* = 1) (60), France (*n* = 1) (24), Italy (*n* = 1) (32), and Switzerland (*n* = 1) (62). The multi-country studies included the “Intermediate Care for Dementia in Europe” project (*n* = 1) (64), covering 16 countries: Bosnia, Croatia, Georgia, Greece, France, Hungary, Ireland, Israel, Italy, Latvia, Poland, Portugal, Romania, Switzerland, the United Kingdom, and Ukraine, and the “Actifcare” project (*n* = 4) (35, 38, 50, 66), involving eight countries: Germany, Ireland, the Netherlands, Norway, Sweden, the United Kingdom, Italy, and Portugal. One study (*n* = 1) (57) involved Germany, Spain, and the United Kingdom, and another study (*n* = 1) (65) involved the United Kingdom, France, and Cyprus, highlighting the collaborative nature of the research.

The methodologies employed in the studies varied significantly. Qualitative studies (*n* = 26) utilized different techniques, including interviews (*n* = 15) (21, 28, 29, 33, 36, 37, 47, 51–54, 56, 59, 61, 62), focus groups (*n* = 3) (39, 42, 45), surveys (*n* = 2) (32), both focus groups and interviews (*n* = 5) (27, 30, 48, 49, 60), and, a combination of surveys and focus groups (*n* = 1) (44). Cross-sectional studies (*n* = 15) were frequent, primarily using surveys (*n* = 10) (23, 25, 40, 41, 50, 55, 58, 63, 64, 67), with some employing interviews (*n* = 4) (22, 31, 34, 46) and one using both focus groups and interviews (*n* = 1) (65). Cohort studies (*n* = 6) demonstrated varied approaches, with some employing surveys (*n* = 4) (26, 35, 38, 66), one combining surveys and healthcare data (*n* = 1) (57), and one utilizing only healthcare data (*n* = 1) (43).

The reviewed studies encompassed various populations, with sample size ranging from 5 to 1,283. The focus of most studies was on caregivers (*n* = 13) (30, 32, 36, 42, 43, 46, 48, 51, 53, 55, 58, 65, 67), while others concentrated solely on HCWs (*n* = 8) (23, 24, 27, 33, 44, 45, 63, 64), and on people living with dementia (*n* = 7) (31, 34, 40, 49, 52, 62, 66). Both caregivers and people living with dementia participated in fourteen studies (21, 22, 25, 26, 28, 29, 35, 38, 41, 47, 50, 56, 57, 59). Some studies (*n* = 4) included both HCWs and caregivers (37, 39, 60, 61), and one study (*n* = 1) included people living with dementia, caregivers, and HCWs (54). Furthermore, five of the previous studies were focused on minority groups (30, 37, 48, 51, 60).

The studies assessed various care settings, focusing on either a single setting or multiple settings. The majority of studies (*n* = 15) focused on home-based care (21, 22, 28, 32, 34, 35, 40, 47, 51, 52, 54–56, 59, 66), community-based care (*n* = 10) (26, 36, 38, 43, 49, 50, 57, 58, 61, 65), primary care (*n* = 3) (24, 31, 33), clinical or hospital-based care (*n* = 3) (25, 29, 44), residential or long-term care facilities (*n* = 1) (63). Furthermore, multiple settings were explored in several studies: for instance, there were studies that combined multiple setting (*n* = 15) (23, 27, 30, 37, 39, 41, 42, 45, 46, 48, 53, 60, 62, 64, 67).

The majority of studies included in the analysis achieved the highest level of quality overall; however, only a small number of articles reached a quality score of 80% based on the MMAT assessment. Refer to Table 3 and Supplementary Table 3 for additional details.

### 3.3 Identified needs

Multiple needs have been identified. These needs were broadly categorized into psychosocial, emotional and social, educational and informational, cultural, healthcare and barriers to care. Additional details are provided in Table 3. The proposed categories did not influence the article analysis and were defined to enhance the presentation of the findings.

#### 3.3.1 Psychosocial and emotional needs

People living with dementia expressed a high need for psychological support due to the significant stress associated with unmet social needs (50), such as activities of daily living, psychological distress, and the need for companionship (41). People living with dementia with hearing and/or visual impairment also expressed the need for psychological support (65). They rated their unmet social needs significantly lower than their caregivers (35), with a negative relationship observed between these unmet needs and both their own and their caregivers’ quality of life (35, 38), as well as the level of neuropsychiatric symptoms over time (26, 66), and demand support for their emotional and psychological well-being (66).

Caregivers expressed more unmet social needs than people living with dementia (41), highlighting the necessity for psychological, emotional, and social support to manage the stress burden associated with caregiving (22, 25, 31, 40, 42, 43, 53). As the disease progressed, the burden on caregivers gradually increased, necessitating enhanced emotional support (57). Caregivers expressed the need for support groups for emotional well-being (54), respite breaks (39), and assistance in managing the behavioral and neuropsychiatric symptoms of the people living with dementia, reducing distress and depressive symptoms, and improving quality of life (67).

During the COVID-19 pandemic, both caregivers and people living with dementia expressed an increased need for psychological support to manage high stress levels due to isolation and the new challenges arising from emerging social and health difficulties (55, 58).

#### 3.3.2 Educational and informational needs

People living with dementia expressed several informational needs, including a general need for in-depth information about dementia (31), demanding online resources that focus on the course of the disease and related issues (62). Those with hearing and/or visual impairments specifically noted a need for more education on the use of assistive devices (65).

Caregivers demonstrated a significant need for information (50) and expressed a strong desire for training and groups to facilitate information sharing (53). Their educational needs cover managing various aspects of dementia, particularly at the beginning of the care process and throughout the care pathway, including special care situations and behavioral problems (42). They require information on dementia progression, disease management, and available care and support options (54). Additionally, caregivers expressed the need for information, awareness, and education on self-care health behaviors (39), and information on technological devices (53).

Additionally, both people living with dementia and their caregivers emphasized the necessity for specific information about the disease and the available support, including social counseling and legal assistance (22), as well as education on medication management and how to address related anxiety (28, 56).

HCWs have numerous educational needs in dementia care. These include training in dementia diagnosis and behavior management (27), as well as education on dementia management, assessment, legal and ethical aspects, interventions, and national dementia strategies (23). Additionally, HCP expressed the need for educational programs related to dementia care, and to have better communication and collaboration within the team (23, 44). General practitioners (GPs) specifically highlighted the need for continuing education and training, pointing out a lack of information and regular updates on the management of intermediate care services in their areas (64). This is particularly important for non-pharmacological management of dementia, effective communication with families and people living with dementia (24), and comprehensive information on disease management (32). Nurses require training in medication and pain management (63), while physiotherapists need further training in dementia and evidence-based physiotherapy guidelines to better understand their role (45).

#### 3.3.3 Cultural needs

People living with dementia sought a reconsideration of public perceptions, emphasizing acknowledgment of their capabilities rather than focusing solely on their disabilities (49) and caregivers expressed the need for more culturally informed care to enhance dementia care (53). GPs expressed a need for cultural competence in society and healthcare to address stigma and improve societal perceptions of dementia (33), as well as to reduce the stigmatization families face when seeking intermediate care (64).

Across various European countries, caregivers from minority ethnic groups have expressed specific needs for cultural sensitivity and competence in dementia care. In the United Kingdom, Bangladeshi caregivers highlighted the need for a deeper understanding of cultural and religious values to improve cultural competence (48). In Belgium, Moroccan caregivers and HCWs emphasized the need for culture-sensitive tests and language support in dementia care (37). In Germany, Turkish caregivers expressed the need for better access to relevant information and the incorporation of Turkish culture into healthcare services (51). Similarly, in Denmark, HCWs and caregivers from Turkish, Pakistani, and Arabic-speaking minority groups require increased cultural sensitivity and competence in healthcare interactions, as well as improved awareness and understanding of dementia within their communities (60). In the Netherlands, caregivers from Turkish, Moroccan, and Surinamese Creole backgrounds need support in managing the emotional and social challenges of caregiving in culturally diverse contexts (30).

#### 3.3.4 Healthcare needs

Healthcare for people living with dementia encompasses a wide range of needs, including nursing care and treatment, drug treatment and care, medical diagnosis and treatment, special therapies, social counseling, and legal support (31). People with severe dementia have a greater need for informal care for activities of daily living, and the need to utilize formal care services increases with the severity of dementia (34), as well as the need for access to healthcare resources (57). Both people living with dementia and their caregivers expressed significant physical and environmental needs, such as medication management, support for physical activity, fall prevention, sensory support, food preparation, personal hygiene, and money management (40, 59, 65).

Family caregivers expressed a strong need for both instrumental and formal support from HCWs to expedite diagnosis and ensure effective treatment (42), as well as financial support (32). The organization and coordination of services are critical to ensuring continuity of support, which plays a crucial role in promoting self-care health behaviors among people living with dementia (53).

HCWs and people living with dementia emphasized the need for a proactive approach to person-centered care (27). Integration of services is particularly necessary, with a focus on providing sensory aids to address the increasing difficulties faced by people living with dementia who also have hearing or visual impairments (65).

#### 3.3.5 Barriers to care

Some needs expressed address barriers to dementia care. These include the need for easier access to care due to the complexity and fragmentation of the care system (54). Informal caregivers express the need to access support services (53), facing logistical barriers in organizing support and care, often involving multiple agencies, professionals, friends, and family members, and find that bureaucracy adds frustration and is time-consuming (36). Caregivers also need support to navigate advanced care planning (21).

People living with dementia face numerous barriers to physical activity due to physical health and cognitive impairment, with dementia progression increasing the need for physical activity while activity levels decrease (29). Caregivers experience difficulties in improving their quality of life, with key barriers including fear of social exclusion and isolation, concerns about the safety of the person they care for, and challenges in maintaining physical activity (29). Lack of time and their caregiving role are significant barriers to timely care (59) and to physical activity (59).

Caregivers also express the need for financial support to cope with the high costs of intermediate care for dementia (51), especially for those living in rural areas due to geographical barriers (64). Both caregivers and people living with dementia have expressed the need for support in navigating advance care planning, with difficulties finding the right time for discussions and a preference for informal plans (21).

HCWs and caregivers recognized emotional barriers to community care access due to reluctance to question authority and fear of stigma, expressing the need to improve access to community-based services for caregivers and user-centered models of care to facilitate better access and improve the quality of care (61). Barriers to intermediate care for dementia include high costs, disorientation, exacerbation of behavioral and psychological symptoms, living in rural areas, and feelings of shame, sadness, and guilt (64).

During the pandemic, access to healthcare and medications became more difficult, with new barriers arising due to the pandemic (47, 55). Families were isolated without assistance, increasing concerns about the resumption of care provision after the pandemic (47, 55).

## 4 Discussion

This systematic review analysed 47 studies published between 2013 and 2023, revealing a complex range of needs expressed by people living with dementia, their caregivers, and HCWs.

The findings highlight several needs, involving different domains of interest including psychosocial and emotional support, educational and information needs, cultural needs, healthcare need, and barriers to care.

The diverse geographic scope of the studies, with a primary focus on the United Kingdom, along with representation from other European countries and some global perspectives, underscores the universal relevance of the issues surrounding dementia care. Indeed, international collaboration and global coordination are crucial for addressing the unequal impact of dementia worldwide (68).

### 4.1 Psychosocial and emotional needs

People living with dementia expressed a high need for psychological support due to the significant stress associated with unmet social needs (41, 50). This need for psychological support (65) is negatively related to both their own and their caregivers’ quality of life (35, 38), as well as the level of neuropsychiatric symptoms over time (26, 66), which are a major predictor of caregiver burden (69, 70), leading to different distress patterns (71–73). Indeed, caregivers expressed even more unmet social needs than people living with dementia (35, 41), expressing the necessity for psychological, emotional, and social support to manage the stress burden associated with caregiving (22, 25, 31, 39, 40, 42, 43, 53, 54), a need for support that increases as the disease progresses, necessitating greater emotional support (57), particularly to manage the behavioral and neuropsychiatric symptoms of people living with dementia (67). Caregivers can develop skills and competence in coping with these symptoms, which can provide relief from negative states when facing people living with dementia demands at different stages (74). Furthermore, non-pharmacological activities, including physical activity, mental activities and music therapy, improve cognition and neuropsychiatric symptoms (75).

### 4.2 Impact of the COVID-19 pandemic

The COVID-19 pandemic caused substantial disruptions in healthcare systems such as a reduction in face to face consultations, an increase in remote consultations and delayed care for elective pocedures (76, 77), further exacerbating symptoms in people living with dementia, compromising their quality of life (78, 79), and increasing the care burden and psychological distress for family caregivers (80–82). Both caregivers and people living with dementia expressed an increased need for psychological support to manage high stress levels due to isolation and new challenges arising from emerging social and health difficulties (55, 58). Social and instrumental support can mediate the effects of caregivers’ stressors, leading to distinct mental reactions (83, 84).

For people living with dementia, access to comprehensive and easily accessible educational resources is essential (22, 31), particularly through online platforms (62), which, along with mass media and smartphones, are among the top sources of information, offering a variety of information independent of time and location (85). For those with sensory disabilities, information about assistive devices is also crucial (65).

### 4.3 Educational and informational needs

Caregivers also expressed the need for educational programs, mainly focused on dementia care (42, 54, 56), in accordance with the literature which shows that the most frequently reported information needs are information about the disease and patient care (86, 87). Furthermore, they wanted detailed guidance on medication management (28, 56), technological devices (53) self-care health practices (39) and a strong desire for training and groups to facilitate information sharing (53). This preference is supported by literature (85) reporting that HCWs are often perceived as lacking adequate training on dementia care services (88) and the information they provide is frequently considered insufficient (89–91). Education and support services can positively impact people living with dementia and their caregivers by enhancing confidence, reducing stress and depression, and improving overall well-being (92–94). However, the effectiveness of educational programs is influenced by various factors, affecting their delivery and outcomes (95).

HCWs also face significant needs, requiring ongoing training and specialized skills to deliver effective and personalized care (23, 24, 27, 32, 63): specific training is essential, such as medication management for nurses (63), evidence-based guidelines for physiotherapists (45) and regular updates on intermediate care management for general practitioners (64). Nonetheless, adequate time for training is crucial (96), yet organizations often struggle with resource constraints, including time, finances, and staff availability, which hinder the effective implementation of training initiatives (97–99).

### 4.4 Cultural aspects of dementia care

In many cultures, dementia is frequently perceived as a shameful condition or a normal part of aging rather than a manageable disease (100). This stigma, driven by a lack of understanding and cultural taboos, discourages families and individuals from seeking help, leading to increased isolation and exacerbating difficulties in managing the disease (101). Such social isolation contributes to higher rates of loneliness and depression, worsening mental health and creating a cycle of exclusion (102).

People living with dementia and caregivers emphasize the need for cultural competence in both societal and healthcare contexts to address this stigma and improve perceptions of dementia (49, 53). Cultural barriers and misconceptions significantly impact families’ experiences and expectations of dementia care, making culturally informed care essential (103). HCWs also recognize the importance of cultural competence, particularly in reducing stigmatization within intermediate care settings (33, 64). Fear of discrimination and social isolation can delay diagnosis and treatment, further hindering access to necessary support services (104).

Addressing these issues requires increasing dementia awareness to combat stigma and challenge the perception of dementia as a normal part of aging (105). HCWs must be trained to address stigma and fears associated with dementia to better support diagnosed individuals and their caregivers (106).

### 4.5 Needs of minority groups

Minority groups express a critical need for deeper cultural and religious understanding in dementia care, including effective language support (37, 48, 51, 60). The lack of cultural sensitivity and adaptation by HCWs often results in inadequate care and limited access for these groups (107, 108). Challenges such as language barriers and insufficient culturally adapted assessment tools exacerbate these issues (109–111). Consequently, individuals from these groups may avoid seeking dementia care, underscoring the need for culturally sensitive approaches and improved access to services for all ethnic groups (112–116). Research highlights the importance of understanding diverse sociocultural factors and tailoring interventions to local contexts, especially in low- and middle-income countries (117–120).

### 4.6 Needs to improve access to dementia care

Dementia management necessitates an integrated approach that addresses various needs, including health and treatment requirements (31, 34) and environmental considerations (40, 59, 65). However, health and care systems are often overly complex and challenging for caregivers to navigate, which can lead to delays in seeking or accessing care and increased stress (121, 122), frequently leading to frustration and overload (123). Caregivers wanted robust support from HCWs and improved coordination of services to ensure continuity of care (32, 42, 53). Fragmentation in the care pathway can result in inconsistencies in the quality and continuity of care (124), significantly increasing healthcare costs (125, 126) and the risk of comorbidity (127–129). A proactive, person-centered approach that integrates services and includes attention to disability aids is crucial for providing effective and personalized care (27, 65). Integrated care systems facilitate rapid response to the assessment and management of needs of people living with dementia, highlighting the urgent need for functional and seamless dementia care pathways (130), encompassing specialized dementia care spaces and ensuring well-coordinated care (131). A crucial aspect is focusing on the specific needs of caregivers, with flexibility and sensitivity being key components for the successful adaptation of care for individuals with AD at different stages (85, 132). This approach promotes the well-being of individuals and ensures continuity across professional boundaries, ultimately improving access to specialized care and minimizing disruptions in care plans (130, 131, 133).

### 4.7 Barriers in dementia care

Several barriers can compromise access to care and the fulfillment of needs, including the complexity and fragmentation of the health and care system (54), difficulties in accessing support services (53), and navigating advanced care planning (21). The involvement of multiple agencies, professionals, and administrative hurdles (36) can exacerbate logistical barriers. Another barrier is the need for improved access to community services and user-centered care to address reluctance to challenge authority, fear of stigma (61), as well as disorientation and feelings of shame and guilt (64). Inequities in healthcare access can compromise adequate care, particularly in rural and deprived areas worldwide (134, 135). Consequently, international efforts are underway to enhance access to healthcare services for dementia care. These efforts aim to address these challenges, reduce stigma, prejudice, inequalities and associated costs, and improve the quality of life for affected families (115, 136–138).

### 4.8 Financial barriers and inequities

Lack of financial support is another barrier, especially due to the high costs of intermediate care, particularly for those living in rural areas (64). Financial constraints can restrict access to vital health and social services and high-quality treatments (109, 139–141), which may result in suboptimal disease management and deteriorating health. Therefore, interventions aimed at reducing financial inequalities could lead to improved health outcomes for older adults (142–144).

### 4.9 Needs to improve quality of life

There are also barriers to improving quality of life, such as a lack of time, social challenges related to physical activities, and the progression of diseases, which exacerbate these issues (29, 59). However, physical activity has a positive effect in mitigating cognitive decline associated with dementia (145, 146). Considering the positive impact of physical activity and the challenges faced by families, it becomes even more important to incentivize programs and initiatives that support and promote physical activity (147, 148).

## 5 Strengths and limitations

To our knowledge, this is the first review to provide an integrated synthesis of the main unmet needs of people living with dementia, their caregivers, and healthcare workers. Whereas most previous studies have examined these groups in isolation, this review considers them collectively, highlighting the interdependent nature of their experiences within dementia care systems and supporting the development of coordinated interventions that reflect the real-world complexity of dementia care. It features several strengths that contribute to the robustness of its conclusions. The review adheres to the Preferred Reporting Items for Systematic Reviews and Meta-Analyses (PRISMA) guidelines, ensuring a transparent and meticulous review process that minimizes bias and enhances reproducibility. A comprehensive search strategy was employed, spanning multiple databases over a decade, to maximize the retrieval of pertinent literature and provide a thorough examination of the topic. The involvement of multiple reviewers bolsters the reliability of study selection and minimizes the likelihood of errors. Additionally, the systematic assessment of the methodological quality of the included studies strengthens the review’s findings by identifying potential biases and limitations within individual studies. The studies included in the review were conducted across various European countries and covered diverse roles, including informal caregivers, HCWs, and people living with dementia, analyzing these groups both collectively and separately to produce robust findings.

However, several limitations need to be acknowledged. One study included in the sample did not exclusively consist of people with a formal diagnosis of dementia. This could limit the generalizability of the findings, as the experiences and needs of individuals without an official diagnosis might differ significantly from those of individuals with diagnosed dementia. Including participants without a diagnosis could introduce variability in the data and affect the accuracy of the conclusions, making it more challenging to identify the specific needs and challenges faced solely by people living with dementia. Most studies did not specify the race and ethnicity of the participants, which limits the understanding of the needs across different racial and ethnic groups. Nonetheless, some studies did address the specific needs of ethnic minorities, providing valuable insights. An additional limitation to consider is the high number of studies conducted exclusively in the UK. However, nearly all findings identified in these studies were also confirmed by research conducted in other European countries. Furthermore, restricting the review to studies published in English and conducted in Europe may introduce language and geographical biases, potentially overlooking valuable insights from non-English literature or studies conducted in other regions. Additionally, reliance on published literature may lead to publication bias, as studies with positive results are more likely to be published, potentially skewing the overall findings. However, the themes identified may be relevant beyond Europe, and could offer useful insights for informing dementia care strategies in other global contexts, particularly in countries facing similar demographic and health system challenges.

## 6 Conclusion

This systematic review underscores the broad range of needs, unmet needs and barriers within dementia care systems in Europe, affecting people living with dementia, caregivers, and healthcare professionals. The findings highlight significant challenges in social inclusion and access to support services for families and people living with dementia, and the need for continuous training for healthcare workers and professionals. The economic impact of unmet also needs to be better understood. There is an urgent need for public policies that enhance support networks, improve resource availability, and promote culturally sensitive care approaches. Future research should focus on the development integrated strategies to better address these needs and ensure a more robust and effective dementia care framework. This could include exploring the role of technology (such as telehealth, assistive devices and online support platforms) in addressing unmet needs.

## Footnotes

^1^ https://www.rayyan.ai
