# Supplementary material for: The needs and unmet needs for people living with dementia, caregivers and care workers in dementia health care systems: a systematic review
Source: Front Public Health. 2025 Aug 20;13:1605993. doi: 10.3389/fpubh.2025.1605993 (PMC12404970; doi:10.3389/fpubh.2025.1605993)
Supplement: Supplementary file 1 [file Table_1.docx]

| **Ref ID** | **Reason for Exclusion** | ***Details on the Reason for Exclusion*** |
| --- | --- | --- |
| [1] | Different population | *Not formal dementia diagnosis* |
| [2] | Different population | *Older/adult, not people living with dementia* |
| [3] | Different population | *Older/adult, not people living with dementia* |
| [4] | Full-text not available |  |
| [5] | Full-text not available |  |
| [6] | Not focused on needs | *Analyse an intervention/therapy* |
| [7] | Not focused on needs | *Analyse an intervention/therapy* |
| [8] | Not focused on needs | *Analyse an intervention/therapy* |
| [9] | Not focused on needs | *Analyse an intervention/therapy* |
| [10] | Not focused on needs | *Analyse an intervention/therapy* |
| [11] | Not focused on needs | *Analyse an intervention/therapy* |
| [12] | Not focused on needs | *Analyse an intervention/therapy* |
| [13] | Not focused on needs | *Analyse an intervention/therapy* |
| [14] | Not focused on needs | *Analyse an intervention/therapy* |
| [15] | Not focused on needs | *Analyse an intervention/therapy* |
| [16] | Not focused on needs | *Analyse an intervention/therapy* |
| [17] | Not focused on needs | *Analyse an intervention/therapy* |
| [18] | Not focused on needs | *Analyse an intervention/therapy* |
| [19] | Not focused on needs | *Analyse an intervention/therapy* |
| [20] | Not focused on needs | *Analyse an intervention/therapy* |
| [21] | Not focused on needs | *Analyse an intervention/therapy* |
| [22] | Not focused on needs | *Analyse an intervention/therapy* |
| [23] | Not focused on needs | *Analyse an intervention/therapy* |
| [24] | Not focused on needs | *Analyse an intervention/therapy* |
| [25] | Not focused on needs | *Analyse an intervention/therapy* |
| [26] | Not focused on needs | *Analyse an intervention/therapy* |
| [27] | Not focused on needs | *Analyse an intervention/therapy* |
| [28] | Not focused on needs | *Analyse an intervention/therapy* |
| [29] | Not focused on needs | *Analyse an intervention/therapy* |
| [30] | Not focused on needs | *Analyse an intervention/therapy* |
| [31] | Not focused on needs | *Analyse an intervention/therapy* |
| [32] | Not focused on needs | *Analyse barrier to care* |
| [33] | Not focused on needs | *Analyse barrier to care* |
| [34] | Not focused on needs | *Analyse barrier to care* |
| [35] | Not focused on needs | *Analyse barrier to care* |
| [36] | Not focused on needs | *Analyse barrier to care* |
| [37] | Not focused on needs | *Analyse barrier to care* |
| [38] | Not focused on needs | *Analyse barrier to care* |
| [39] | Not focused on needs | *Analyse barrier to care* |
| [40] | Not focused on needs | *Analyse barrier to care* |
| [41] | Not focused on needs | *Analyse barrier to care* |
| [42] | Not focused on needs | *Analyse barrier to care* |
| [43] | Not focused on needs | *Analyse barrier to care* |
| [44] | Not focused on needs | *Analyse barrier to care* |
| [45] | Not focused on needs | *Analyse barrier to care* |
| [46] | Not focused on needs | *Analyse barrier to care* |
| [47] | Not focused on needs | *Analyse barrier to care* |
| [48] | Not focused on needs | *Analyse barrier to care* |
| [49] | Not focused on needs | *Analyse barrier to care* |
| [50] | Not focused on needs | *Analyse barrier to care* |
| [51] | Not focused on needs | *Analyse barrier to care* |
| [52] | Not focused on needs | *Analyse barrier to care* |
| [53] | Not focused on needs | *Analysis of Healthcare organisation* |
| [54] | Not focused on needs | *Analysis of Healthcare organisation* |
| [55] | Not focused on needs | *Analysis of Healthcare organisation* |
| [56] | Not focused on needs | *Analysis of Healthcare organisation* |
| [57] | Not focused on needs | *Analysis of Healthcare organisation* |
| [58] | Not focused on needs | *Analysis of Healthcare organisation* |
| [59] | Not focused on needs | *Analysis of Healthcare organisation* |
| [60] | Not focused on needs | *Analysis of Healthcare organisation* |
| [61] | Not focused on needs | *Analysis of Healthcare organisation* |
| [62] | Not focused on needs | *Analysis of Healthcare organisation* |
| [63] | Not focused on needs | *Analysis of Healthcare organisation* |
| [64] | Not focused on needs | *Analysis of Healthcare organisation* |
| [65] | Not focused on needs | *Analysis of Healthcare organisation* |
| [66] | Not focused on needs | *Comparision between population* |
| [67] | Not focused on needs | *Diagnosis evaluation* |
| [68] | Not focused on needs | *Epidemiological study* |
| [69] | Not focused on needs | *Epidemiological study* |
| [70] | Not focused on needs | *Epidemiological study* |
| [71] | Not focused on needs | *Epidemiological study* |
| [72] | Not focused on needs | *Epidemiological study* |
| [73] | Not focused on needs | *Epidemiological study* |
| [74] | Not focused on needs | *Epidemiological study* |
| [75] | Not focused on needs | *Epidemiological study* |
| [76] | Not focused on needs | *Epidemiological study* |
| [77] | Not focused on needs | *Expert opininion on healthcare setting* |
| [78] | Not focused on needs | *Expert opininion on healthcare setting* |
| [79] | Not focused on needs | *Focused on legal/Ethical issues* |
| [80] | Not focused on needs | *Focused on mental health* |
| [81] | Not focused on needs | *Focused on technology in healthcare* |
| [82] | Not focused on needs | *Focused on technology in healthcare* |
| [83] | Not focused on needs | *Focused on technology in healthcare* |
| [84] | Not focused on needs | *Focused on technology in healthcare* |
| [85] | Not focused on needs | *Focused on technology in healthcare* |
| [86] | Not focused on needs | *Focused on technology in healthcare* |
| [87] | Not focused on needs | *Focused on technology in healthcare* |
| [88] | Not focused on needs | *Focused on technology in healthcare* |
| [89] | Not focused on needs | *Focused on technology in healthcare* |
| [90] | Not focused on needs | *Focused on technology in healthcare* |
| [91] | Not focused on needs | *Healthcare efficacy/efficiency evaluation* |
| [92] | Not focused on needs | *Healthcare efficacy/efficiency evaluation* |
| [93] | Not focused on needs | *Healthcare efficacy/efficiency evaluation* |
| [94] | Not focused on needs | *Healthcare efficacy/efficiency evaluation* |
| [95] | Not focused on needs | *Methodology in research* |
| [96] | Not focused on needs | *Study of pandemic effect* |
| [97] | Not focused on needs | *Study of pandemic effect* |
| [98] | Not set in a European nation |  |
| [99] | Not set in a European nation |  |
| [100] | Not set in a European nation |  |
| [101] | Not set in a European nation |  |
| [102] | Not set in a European nation |  |
| [103] | Not set in a European nation |  |
| [104] | Not set in a European nation |  |
| [105] | Not set in a European nation |  |
| [106] | Study design/type | *Consensus* |
| [107] | Study design/type | *Protocol study* |
| [108] | Study design/type | *Review* |
| [109] | Study design/type | *Review* |
| [110] | Study design/type | *Review* |
| [111] | Study design/type | *Review* |
| [112] | Study design/type | *Review* |
| [113] | Study design/type | *Review* |
| [114] | Study design/type | *Review* |
| [115] | Study design/type | *Review* |
| [116] | Study design/type | *Review* |
| [117] | Study design/type | *Review* |
| [118] | Study design/type | *Review* |
| [119] | Study design/type | *Review* |
| [120] | Study design/type | *Review* |
| [121] | Study design/type | *Review* |
| [122] | Study design/type | *Review* |
| [123] | Study design/type | *Review* |
| [124] | Study design/type | *Review* |
| [125] | Study design/type | *Review* |
| [126] | Study design/type | *Review* |
| [127] | Study design/type | *Review* |
| [128] | Study design/type | *Review* |
| [129] | Study design/type | *Trial* |
| [130] | Study design/type | *Validation* |

**Supplementary Table 1**. Reasons for exclusion during full-text analysis.

**References**

1. Wesselman LMP, Schild AK, Coll-Padros N, et al. Wishes and preferences for an online lifestyle program for brain health-A mixed methods study. *Alzheimers Dement (N Y)*. 2018;4(1):141-149. doi:10.1016/j.trci.2018.03.003

2. Rantakokko M, Portegijs E, Viljanen A, Iwarsson S, Rantanen T. Task Modifications in Walking Postpone Decline in Life-Space Mobility Among Community-Dwelling Older People: A 2-year Follow-up Study. *J Gerontol A Biol Sci Med Sci*. Sep 1 2017;72(9):1252-1256. doi:10.1093/gerona/glw348

3. Neter E, Chachashvili-Bolotin S, Erlich B, Ifrah K. Benefiting From Digital Use: Prospective Association of Internet Use With Knowledge and Preventive Behaviors Related to Alzheimer Disease in the Israeli Survey of Aging. *JMIR Aging*. Apr 30 2021;4(2):e25706. doi:10.2196/25706

4. Hirt J, Burgstaller M, Zeller A, Beer T. Needs of people with dementia and their informal caregivers concerning assistive technologies. *Pflege*. 2019;32(6):295-304. doi:10.1024/1012-5302/a000682

5. Monin JK, Jorgensen TD, MacNeil Vroomen JL. Self-Reports and Caregivers' Proxy Reports of Unmet Needs of Persons With Dementia: Implications for Both Partners' Health-Related Quality of Life. *Am J Geriatr Psychiatry*. Mar 2020;28(3):363-367. doi:10.1016/j.jagp.2019.10.006

6. Hallberg IR, Cabrera E, Jolley D, et al. Professional care providers in dementia care in eight European countries; their training and involvement in early dementia stage and in home care. *Dementia (London)*. Sep 2016;15(5):931-57. doi:10.1177/1471301214548520

7. Dr Grant Gibson DJR, Catherine Pemble, Rog Harrison, Kim Strachan, Sheila Thorburn. *Dementia Friendly Walking Project*. Vol. 2024. 2017. *Dementia Friendly Walking paths for all*. <https://www.pathsforall.org.uk/mediaLibrary/other/english/dementiafriendlyevaluationexecutivesummary2017.pdf>

8. Malinowsky C, Nygard L, Kottorp A. Using a screening tool to evaluate potential use of e-health services for older people with and without cognitive impairment. *Aging Ment Health*. 2014;18(3):340-5. doi:10.1080/13607863.2013.832731

9. Zwingmann I, Dreier-Wolfgramm A, Esser A, et al. Why do family dementia caregivers reject caregiver support services? Analyzing types of rejection and associated health-impairments in a cluster-randomized controlled intervention trial. *BMC Health Serv Res*. Feb 14 2020;20(1):121. doi:10.1186/s12913-020-4970-8

10. Bantry White E, Montgomery P. Supporting people with dementia to walkabout safely outdoors: development of a structured model of assessment. *Health Soc Care Community*. Jul 2016;24(4):473-84. doi:10.1111/hsc.12226

11. De Poli C, Oyebode J, Airoldi M, Glover R. A need-based, multi-level, cross-sectoral framework to explain variations in satisfaction of care needs among people living with dementia. *BMC Health Serv Res*. Jul 15 2020;20(1):657. doi:10.1186/s12913-020-05416-x

12. Videla L, Benejam B, Pegueroles J, et al. Longitudinal Clinical and Cognitive Changes Along the Alzheimer Disease Continuum in Down Syndrome. *JAMA Netw Open*. Aug 1 2022;5(8):e2225573. doi:10.1001/jamanetworkopen.2022.25573

13. Bogolepova AN, Brovko EV, Gavrilova SI, et al. Roadmap for ecosystem-based approach for patients with Alzheimer disease in Russia: current needs, barriers, and possible solutions (resolution of the scientific and practical experts meeting). *Zh Nevrol Psikhiatr Im S S Korsakova*. 2022;122(7):121-131. Dorozhnaya karta po okazaniyu pomoshchi patsientu s bolezn'yu Al'tsgeimera v Rossii v formate ekosistemy: tekushchie potrebnosti, bar'ery i vozmozhnye resheniya (rezolyutsiya nauchno-prakticheskoi vstrechi ekspertov). doi:10.17116/jnevro2022122071121

14. Bray J, Evans S, Thompson R, et al. Understanding the needs of people with dementia and family carers. *Nurs Older People*. Sep 2015;27(7):18, 20-3. doi:10.7748/nop.27.7.18.e699

15. Cedervall Y, Torres S, Aberg AC. Maintaining well-being and selfhood through physical activity: experiences of people with mild Alzheimer's disease. *Aging Ment Health*. 2015;19(8):679-88. doi:10.1080/13607863.2014.962004

16. Mazurek J, Szczesniak D, Lion KM, Droes RM, Karczewski M, Rymaszewska J. Does the Meeting Centres Support Programme reduce unmet care needs of community-dwelling older people with dementia? A controlled, 6-month follow-up Polish study. *Clin Interv Aging*. 2019;14:113-122. doi:10.2147/CIA.S185683

17. van Rijn A, Meiland F, Droes RM. Linking DemenTalent to Meeting Centers for people with dementia and their caregivers: a process analysis into facilitators and barriers in 12 Dutch Meeting Centers. *Int Psychogeriatr*. Oct 2019;31(10):1433-1445. doi:10.1017/S1041610219001108

18. Samsi K, Abley C, Campbell S, et al. Negotiating a labyrinth: experiences of assessment and diagnostic journey in cognitive impairment and dementia. *Int J Geriatr Psychiatry*. Jan 2014;29(1):58-67. doi:10.1002/gps.3969

19. Charlesworth G, Burnell K, Crellin N, et al. Peer support and reminiscence therapy for people with dementia and their family carers: a factorial pragmatic randomised trial. *J Neurol Neurosurg Psychiatry*. Nov 2016;87(11):1218-1228. doi:10.1136/jnnp-2016-313736

20. Laakkonen ML, Kautiainen H, Holtta E, et al. Effects of Self-Management Groups for People with Dementia and Their Spouses--Randomized Controlled Trial. *J Am Geriatr Soc*. Apr 2016;64(4):752-60. doi:10.1111/jgs.14055

21. Prick AE, de Lange J, Twisk J, Pot AM. The effects of a multi-component dyadic intervention on the psychological distress of family caregivers providing care to people with dementia: a randomized controlled trial. *Int Psychogeriatr*. Dec 2015;27(12):2031-44. doi:10.1017/S104161021500071X

22. Quinn C, Toms G, Jones C, et al. A pilot randomized controlled trial of a self-management group intervention for people with early-stage dementia (The SMART study). *Int Psychogeriatr*. May 2016;28(5):787-800. doi:10.1017/S1041610215002094

23. Joling KJ, Bosmans JE, van Marwijk HW, et al. The cost-effectiveness of a family meetings intervention to prevent depression and anxiety in family caregivers of patients with dementia: a randomized trial. *Trials*. Sep 22 2013;14(1):305. doi:10.1186/1745-6215-14-305

24. Cove J, Jacobi N, Donovan H, Orrell M, Stott J, Spector A. Effectiveness of weekly cognitive stimulation therapy for people with dementia and the additional impact of enhancing cognitive stimulation therapy with a carer training program. *Clin Interv Aging*. 2014;9:2143-50. doi:10.2147/CIA.S66232

25. Hoel V, Seibert K, Domhoff D, et al. Social Health among German Nursing Home Residents with Dementia during the COVID-19 Pandemic, and the Role of Technology to Promote Social Participation. *Int J Environ Res Public Health*. Feb 10 2022;19(4)doi:10.3390/ijerph19041956

26. Budak KB, Laporte Uribe F, Meiland F, et al. Implementing Active Assisted Living Technology in the Long-term Care of People Living With Dementia to Address Loneliness: European Survey. *JMIR Aging*. Jun 14 2023;6:e45231. doi:10.2196/45231

27. Soderhamn U, Landmark B, Eriksen S, Soderhamn O. Participation in physical and social activities among home-dwelling persons with dementia - experiences of next of kin. *Psychol Res Behav Manag*. 2013;6:29-36. doi:10.2147/PRBM.S46334

28. Finnanger Garshol B, Ellingsen-Dalskau LH, Pedersen I. Physical activity in people with dementia attending farm-based dementia day care - a comparative actigraphy study. *BMC Geriatr*. Jun 22 2020;20(1):219. doi:10.1186/s12877-020-01618-4

29. Diaz A, Gove D, Nelson M, et al. Conducting public involvement in dementia research: The contribution of the European Working Group of People with Dementia to the ROADMAP project. *Health Expect*. Jun 2021;24(3):757-765. doi:10.1111/hex.13246

30. Jimenez S, Bueno B, Navarro AB. Do the caregiving spouses of people with dementia in Spain perceive the same barriers for taking part in interventions as caregiving offspring? *Health Soc Care Community*. Sep 2022;30(5):e2385-e2394. doi:10.1111/hsc.13678

31. Bleijlevens MH, Stolt M, Stephan A, et al. Changes in caregiver burden and health-related quality of life of informal caregivers of older people with Dementia: evidence from the European RightTimePlaceCare prospective cohort study. *J Adv Nurs*. Jun 2015;71(6):1378-91. doi:10.1111/jan.12561

32. Balsinha C, Iliffe S, Dias S, Freitas A, Barreiros FF, Goncalves-Pereira M. Dementia and primary care teams: obstacles to the implementation of Portugal's Dementia Strategy. *Prim Health Care Res Dev*. Feb 18 2022;23:e10. doi:10.1017/S1463423621000876

33. Burgon C, Darby J, Pollock K, et al. Perspectives of healthcare professionals in England on falls interventions for people with dementia: a qualitative interview study. *BMJ Open*. Feb 11 2019;9(2):e025702. doi:10.1136/bmjopen-2018-025702

34. Czapka EA, Sagbakken M. "It is always me against the Norwegian system." barriers and facilitators in accessing and using dementia care by minority ethnic groups in Norway: a qualitative study. *BMC Health Serv Res*. Oct 15 2020;20(1):954. doi:10.1186/s12913-020-05801-6

35. Giebel C, Sutcliffe C, Darlington-Pollock F, et al. Health Inequities in the Care Pathways for People Living with Young- and Late-Onset Dementia: From Pre-COVID-19 to Early Pandemic. *Int J Environ Res Public Health*. Jan 14 2021;18(2)doi:10.3390/ijerph18020686

36. Giebel C, Lord K, Cooper C, et al. A UK survey of COVID-19 related social support closures and their effects on older people, people with dementia, and carers. *Int J Geriatr Psychiatry*. Mar 2021;36(3):393-402. doi:10.1002/gps.5434

37. Giebel C, Robertson S, Beaulen A, Zwakhalen S, Allen D, Verbeek H. "Nobody Seems to Know Where to Even Turn To": Barriers in Accessing and Utilising Dementia Care Services in England and The Netherlands. *Int J Environ Res Public Health*. Nov 22 2021;18(22)doi:10.3390/ijerph182212233

38. Hirt J, Karrer M, Adlbrecht L, Saxer S, Zeller A. Facilitators and barriers to implement nurse-led interventions in long-term dementia care: a qualitative interview study with Swiss nursing experts and managers. *BMC Geriatr*. Mar 5 2021;21(1):159. doi:10.1186/s12877-021-02120-1

39. Jutlla K. The impact of migration experiences and migration identities on the experiences of services and caring for a family member with dementia for Sikhs living in Wolverhampton, UK. *Ageing and Society*. 2014;35(5):1032-1054. doi:10.1017/s0144686x14000658

40. Kerpershoek L, Wolfs C, Verhey F, et al. Optimizing access to and use of formal dementia care: Qualitative findings from the European Actifcare study. *Health Soc Care Community*. Sep 2019;27(5):e814-e823. doi:10.1111/hsc.12804

41. Kulmala J, Rosenberg A, Ngandu T, et al. Facilitators and barriers to implementing lifestyle intervention programme to prevent cognitive decline. *Eur J Public Health*. Oct 11 2021;31(4):816-822. doi:10.1093/eurpub/ckab087

42. Kupeli N, Leavey G, Harrington J, et al. What are the barriers to care integration for those at the advanced stages of dementia living in care homes in the UK? Health care professional perspective. *Dementia (London)*. Feb 2018;17(2):164-179. doi:10.1177/1471301216636302

43. Mariani E, Vernooij-Dassen M, Koopmans R, Engels Y, Chattat R. Shared decision-making in dementia care planning: barriers and facilitators in two European countries. *Aging Ment Health*. Jan 2017;21(1):31-39. doi:10.1080/13607863.2016.1255715

44. Midtbust MH, Alnes RE, Gjengedal E, Lykkeslet E. Perceived barriers and facilitators in providing palliative care for people with severe dementia: the healthcare professionals' experiences. *BMC Health Serv Res*. Sep 12 2018;18(1):709. doi:10.1186/s12913-018-3515-x

45. Nielsen TR, Nielsen DS, Waldemar G. Barriers to post-diagnostic care and support in minority ethnic communities: A survey of Danish primary care dementia coordinators. *Dementia (London)*. Nov 2020;19(8):2702-2713. doi:10.1177/1471301219853945

46. Sriram V, Jenkinson C, Peters M. Impact of COVID-19 restrictions on carers of persons with dementia in the UK: a qualitative study. *Age Ageing*. Nov 10 2021;50(6):1876-1885. doi:10.1093/ageing/afab156

47. Stephan A, Bieber A, Hopper L, et al. Barriers and facilitators to the access to and use of formal dementia care: findings of a focus group study with people with dementia, informal carers and health and social care professionals in eight European countries. *BMC Geriatr*. Jun 4 2018;18(1):131. doi:10.1186/s12877-018-0816-1

48. Stephan A, Mohler R, Renom-Guiteras A, Meyer G. Successful collaboration in dementia care from the perspectives of healthcare professionals and informal carers in Germany: results from a focus group study. *BMC Health Serv Res*. May 28 2015;15(1):208. doi:10.1186/s12913-015-0875-3

49. Gorska S, Forsyth K, Irvine L, et al. Service-related needs of older people with dementia: perspectives of service users and their unpaid carers. *Int Psychogeriatr*. Jul 2013;25(7):1107-14. doi:10.1017/S1041610213000343

50. Wheatley A, Bamford C, Brunskill G, Booi L, Dening KH, Robinson L. Implementing post-diagnostic support for people living with dementia in England: a qualitative study of barriers and strategies used to address these in practice. *Age Ageing*. Nov 10 2021;50(6):2230-2237. doi:10.1093/ageing/afab114

51. Kontrimiene A, Blazeviciene A, Liseckiene I, Raila G, Valius L, Jaruseviciene L. Partnership between Primary Health and Social Care Services in the Long-Term Care of Older People with Dementia: A Vignette Study. *Inquiry*. Jan-Dec 2021;58:469580211011933. doi:10.1177/00469580211011933

52. Bieber A, Stephan A, Verbeek H, et al. Access to community care for people with dementia and their informal carers : Case vignettes for a European comparison of structures and common pathways to formal care. *Z Gerontol Geriatr*. Jul 2018;51(5):530-536. Zugang zu professioneller Unterstutzung fur Menschen mit Demenz und ihre Angehorigen : Fallvignetten fur den europaischen Vergleich von Strukturen und Zugangswegen zu professioneller Pflege. doi:10.1007/s00391-017-1266-7

53. O'Shea E, Timmons S, O'Shea E, Irving K. Multiple Stakeholders' Perspectives on Respite Service Access for People With Dementia and Their Carers. *Gerontologist*. Sep 17 2019;59(5):e490-e500. doi:10.1093/geront/gnz095

54. Stein J, Pabst A, Luck T, et al. Unmet Care Needs in the Oldest Old Primary Care Patients with Cognitive Disorders: Results of the AgeCoDe and AgeQualiDe Study. *Dement Geriatr Cogn Disord*. 2017;44(1-2):71-83. doi:10.1159/000478850

55. Haikio K, Cloutier D, Rugkasa J. Is health literacy of family carers associated with carer burden, quality of life, and time spent on informal care for older persons living with dementia? *PLoS One*. 2020;15(11):e0241982. doi:10.1371/journal.pone.0241982

56. Wubbeler M, Thyrian JR, Michalowsky B, et al. How do people with dementia utilise primary care physicians and specialists within dementia networks? Results of the Dementia Networks in Germany (DemNet-D) study. *Health Soc Care Community*. Jan 2017;25(1):285-294. doi:10.1111/hsc.12315

57. Davies N, Walker N, Hopwood J, Iliffe S, Rait G, Walters K. A "separation of worlds": The support and social networks of family carers of people with dementia at the end of life, and the possible role of the internet. *Health Soc Care Community*. Jul 2019;27(4):e223-e232. doi:10.1111/hsc.12701

58. Larsen LS, Normann HK, Hamran T. Collaboration between Sami and non-Sami formal and family caregivers in rural municipalities. *Ethnic and Racial Studies*. 2015;39(5):821-839. doi:10.1080/01419870.2015.1080382

59. NÆSs A, Moen B. Dementia and migration: Pakistani immigrants in the Norwegian welfare state. *Ageing and Society*. 2014;35(8):1713-1738. doi:10.1017/s0144686x14000488

60. Rees JL, Burton A, Walters KR, et al. Exploring how people with dementia can be best supported to manage long-term conditions: a qualitative study of stakeholder perspectives. *BMJ Open*. Oct 8 2020;10(10):e041873. doi:10.1136/bmjopen-2020-041873

61. Thyrian JR, Michalowsky B, Hertel J, et al. How Does Utilization of Health Care Services Change in People with Dementia Served by Dementia Care Networks? Results of the Longitudinal, Observational DemNet-D-Study. *J Alzheimers Dis*. 2018;66(4):1609-1617. doi:10.3233/JAD-180758

62. Davies N, Maio L, Vedavanam K, et al. Barriers to the provision of high-quality palliative care for people with dementia in England: a qualitative study of professionals' experiences. *Health Soc Care Community*. Jul 2014;22(4):386-94. doi:10.1111/hsc.12094

63. Surr CA, Parveen S, Smith SJ, et al. The barriers and facilitators to implementing dementia education and training in health and social care services: a mixed-methods study. *BMC Health Serv Res*. Jun 5 2020;20(1):512. doi:10.1186/s12913-020-05382-4

64. Haaksma ML, O'Driscoll C, Joling KJ, et al. Evaluating the feasibility, experiences, facilitators of and barriers to carers and volunteers delivering Namaste Care to people with dementia in their own home: a qualitative interview study in the UK and the Netherlands. *BMJ Open*. Nov 14 2022;12(11):e063422. doi:10.1136/bmjopen-2022-063422

65. Scalmana S, Di Napoli A, Franco F, et al. Use of health and social care services in a cohort of Italian dementia patients. *Funct Neurol*. Oct-Dec 2013;28(4):265-73.

66. van der Ploeg ES, Bax D, Boorsma M, Nijpels G, van Hout HP. A cross-sectional study to compare care needs of individuals with and without dementia in residential homes in the Netherlands. *BMC Geriatr*. May 24 2013;13(1):51. doi:10.1186/1471-2318-13-51

67. Chithiramohan A, Iliffe S, Khattak I. Identifying barriers to diagnosing dementia following incentivisation and policy pressures: General practitioners' perspectives. *Dementia (London)*. Feb 2019;18(2):514-529. doi:10.1177/1471301216682625

68. Franzen S, Eikelboom WS, van den Berg E, Jiskoot LC, van Hemmen J, Papma JM. Caregiver Burden in a Culturally Diverse Memory Clinic Population: The Caregiver Strain Index-Expanded. *Dement Geriatr Cogn Disord*. 2021;50(4):333-340. doi:10.1159/000519617

69. Axmon A, Karlsson B, Ahlstrom G. Health care utilisation among older persons with intellectual disability and dementia: a registry study. *J Intellect Disabil Res*. Dec 2016;60(12):1165-1177. doi:10.1111/jir.12338

70. Huis In Het Veld J, Verkaik R, van Meijel B, et al. Self-management by family caregivers to manage changes in the behavior and mood of their relative with dementia: an online focus group study. *BMC Geriatr*. May 3 2016;16(1):95. doi:10.1186/s12877-016-0268-4

71. Tan B, Fox S, Kruger C, Lynch M, Shanagher D, Timmons S. Investigating the healthcare utilisation and other support needs of people with young-onset dementia. *Maturitas*. Apr 2019;122:31-34. doi:10.1016/j.maturitas.2019.01.003

72. Hansen A, Hauge S, Bergland A. Meeting psychosocial needs for persons with dementia in home care services - a qualitative study of different perceptions and practices among health care providers. *BMC Geriatr*. Sep 11 2017;17(1):211. doi:10.1186/s12877-017-0612-3

73. Sagbakken M, Spilker RS, Nielsen TR. Dementia and immigrant groups: a qualitative study of challenges related to identifying, assessing, and diagnosing dementia. *BMC Health Serv Res*. Nov 29 2018;18(1):910. doi:10.1186/s12913-018-3720-7

74. Moholt JM, Friborg O, Blix BH, Henriksen N. Factors affecting the use of home-based services and out-of-home respite care services: A survey of family caregivers for older persons with dementia in Northern Norway. *Dementia (London)*. Jul 2020;19(5):1712-1731. doi:10.1177/1471301218804981

75. Pierse T, Keogh F, O'Shea E, Cullinan J. Geographic availability and accessibility of day care services for people with dementia in Ireland. *BMC Health Serv Res*. May 27 2020;20(1):476. doi:10.1186/s12913-020-05341-z

76. Surr CA, Sass C, Burnley N, et al. Components of impactful dementia training for general hospital staff: a collective case study. *Aging Ment Health*. Mar 2020;24(3):511-521. doi:10.1080/13607863.2018.1531382

77. Saks K, Tiit EM, Verbeek H, et al. Most appropriate placement for people with dementia: individual experts' vs. expert groups' decisions in eight European countries. *J Adv Nurs*. Jun 2015;71(6):1363-77. doi:10.1111/jan.12544

78. Mangili S, Ciaffoni L, Sun T, Brambilla A, Capolongo S. Evaluation of stakeholder opinion about Long Term Care Facilities for People with Dementia perceived quality: a web-based survey in the Italian context. *Acta Biomed*. Aug 30 2023;94(S3):e2023143. doi:10.23750/abm.v94iS3.14283

79. Garcia-Ptacek S, Dahlrup B, Edlund AK, Wijk H, Eriksdotter M. The caregiving phenomenon and caregiver participation in dementia. *Scand J Caring Sci*. Jun 2019;33(2):255-265. doi:10.1111/scs.12627

80. Romero-Moreno R, Losada A, Marquez M, et al. Leisure, gender, and kinship in dementia caregiving: psychological vulnerability of caregiving daughters with feelings of guilt. *J Gerontol B Psychol Sci Soc Sci*. Jul 2014;69(4):502-13. doi:10.1093/geronb/gbt027

81. Newton L, Dickinson C, Gibson G, Brittain K, Robinson L. Exploring the views of GPs, people with dementia and their carers on assistive technology: a qualitative study. *BMJ Open*. May 13 2016;6(5):e011132. doi:10.1136/bmjopen-2016-011132

82. Gibson G, Dickinson C, Brittain K, Robinson L. The everyday use of assistive technology by people with dementia and their family carers: a qualitative study. *BMC Geriatr*. Jul 24 2015;15(1):89. doi:10.1186/s12877-015-0091-3

83. van Gils AM, Visser LN, Hendriksen HM, et al. Assessing the Views of Professionals, Patients, and Care Partners Concerning the Use of Computer Tools in Memory Clinics: International Survey Study. *JMIR Form Res*. Dec 3 2021;5(12):e31053. doi:10.2196/31053

84. Hall A, Wilson CB, Stanmore E, Todd C. Implementing monitoring technologies in care homes for people with dementia: A qualitative exploration using Normalization Process Theory. *Int J Nurs Stud*. Jul 2017;72:60-70. doi:10.1016/j.ijnurstu.2017.04.008

85. Chirico I, Giebel C, Lion K, et al. Use of technology by people with dementia and informal carers during COVID-19: A cross-country comparison. *Int J Geriatr Psychiatry*. Sep 2022;37(9)doi:10.1002/gps.5801

86. Tsertsidis A. Challenges in the provision of digital technologies to elderly with dementia to support ageing in place: a case study of a Swedish municipality. *Disabil Rehabil Assist Technol*. Oct 2021;16(7):758-768. doi:10.1080/17483107.2019.1710774

87. Holthe T, Jentoft R, Arntzen C, Thorsen K. Benefits and burdens: family caregivers' experiences of assistive technology (AT) in everyday life with persons with young-onset dementia (YOD). *Disabil Rehabil Assist Technol*. Nov 2018;13(8):754-762. doi:10.1080/17483107.2017.1373151

88. Zygouris S, Gkioka M, Moraitou D, et al. Assessing the Attitudes of Greek Nurses Toward Computerized Dementia Screening. *J Alzheimers Dis*. 2020;78(4):1575-1583. doi:10.3233/JAD-200666

89. Eichler T, Thyrian JR, Fredrich D, et al. The benefits of implementing a computerized intervention-management-system (IMS) on delivering integrated dementia care in the primary care setting. *Int Psychogeriatr*. Aug 2014;26(8):1377-85. doi:10.1017/S1041610214000830

90. Freiesleben SD, Megges H, Herrmann C, Wessel L, Peters O. Overcoming barriers to the adoption of locating technologies in dementia care: a multi-stakeholder focus group study. *BMC Geriatr*. Jun 21 2021;21(1):378. doi:10.1186/s12877-021-02323-6

91. Krutter S, Schaffler-Schaden D, Essl-Maurer R, Seymer A, Osterbrink J, Flamm M. Home care nursing for persons with dementia from a family caregivers' point of view: Predictors of utilisation in a rural setting in Austria. *Health Soc Care Community*. Jan 2022;30(1):389-399. doi:10.1111/hsc.13412

92. Fragkiadaki S, Kontaxopoulou D, Stanitsa E, et al. How Well Did the Healthcare System Respond to the Healthcare Needs of Older People with and without Dementia during the COVID-19 Pandemic? The Perception of Healthcare Providers and Older People from the SI4CARE Project in the ADRION Region. *Geriatrics (Basel)*. Feb 1 2023;8(1)doi:10.3390/geriatrics8010021

93. Ahmad M, van den Broeke J, Saharso S, Tonkens E. Persons With a Migration Background Caring for a Family Member With Dementia: Challenges to Shared Care. *Gerontologist*. Feb 24 2020;60(2):340-349. doi:10.1093/geront/gnz161

94. Caprioli T, Giebel C, Reilly S, Tetlow H, Limbert S, Lloyd-Williams M. Social support services for dementia during the COVID-19 pandemic: A longitudinal survey exploring service adaptations in the United Kingdom. *Health Expect*. Aug 2023;26(4):1726-1737. doi:10.1111/hex.13784

95. Waite J, Poland F, Charlesworth G. Facilitators and barriers to co-research by people with dementia and academic researchers: Findings from a qualitative study. *Health Expect*. Aug 2019;22(4):761-771. doi:10.1111/hex.12891

96. Garcia Santelesforo R, Rodriguez Del Rey T, Perez-Saez E, Pelaez Hernandez B. Impact of confinement measures due to the COVID-19 pandemic on people living with dementia and their caregivers in Spain. *Health Soc Care Community*. Nov 2022;30(6):e5391-e5400. doi:10.1111/hsc.13960

97. Tuijt R, Rait G, Frost R, Wilcock J, Manthorpe J, Walters K. Remote primary care consultations for people living with dementia during the COVID-19 pandemic: experiences of people living with dementia and their carers. *Br J Gen Pract*. Aug 2021;71(709):e574-e582. doi:10.3399/BJGP.2020.1094

98. Ducharme F, Kergoat MJ, Coulombe R, Levesque L, Antoine P, Pasquier F. Unmet support needs of early-onset dementia family caregivers: a mixed-design study. *BMC Nurs*. 2014;13(1):49. doi:10.1186/s12912-014-0049-3

99. Black BS, Johnston D, Rabins PV, Morrison A, Lyketsos C, Samus QM. Unmet needs of community-residing persons with dementia and their informal caregivers: findings from the maximizing independence at home study. *J Am Geriatr Soc*. Dec 2013;61(12):2087-2095. doi:10.1111/jgs.12549

100. Nichols KR, Fam D, Cook C, et al. When dementia is in the house: needs assessment survey for young caregivers. *Can J Neurol Sci*. Jan 2013;40(1):21-8. doi:10.1017/s0317167100012907

101. du Toit SHJ, Baldassar L, Raber CL, et al. Embracing Cultural Diversity - Leadership Perspectives on Championing Meaningful Engagement for Residents Living with Advanced Dementia. *J Cross Cult Gerontol*. Mar 2020;35(1):49-67. doi:10.1007/s10823-019-09387-3

102. Roach P, Drummond N. 'It's nice to have something to do': early-onset dementia and maintaining purposeful activity. *J Psychiatr Ment Health Nurs*. Dec 2014;21(10):889-95. doi:10.1111/jpm.12154

103. Ducharme F, Kergoat MJ, Antoine P, Pasquier F, Coulombe R. The unique experience of spouses in early-onset dementia. *Am J Alzheimers Dis Other Demen*. Sep 2013;28(6):634-41. doi:10.1177/1533317513494443

104. Ketchum FB, Monsees J, Kim AJ, et al. Pathways of care: a qualitative study of barriers and facilitators to engaging dementia care among underserved and minority populations in the US and Germany. *Aging Ment Health*. Feb 2023;27(2):389-398. doi:10.1080/13607863.2022.2033695

105. Roberts E, Shehadeh A. Community Visioning for Innovation in Integrated Dementia Care: Stakeholder Focus Group Outcomes. *J Prim Care Community Health*. Jan-Dec 2021;12:21501327211042791. doi:10.1177/21501327211042791

106. Carter JE, Oyebode JR, Koopmans R. Young-onset dementia and the need for specialist care: a national and international perspective. *Aging Ment Health*. Apr 2018;22(4):468-473. doi:10.1080/13607863.2016.1257563

107. Klein OA, Boekholt M, Afrin D, et al. Effectiveness of a digitally supported care management programme to reduce unmet needs of family caregivers of people with dementia: study protocol for a cluster randomised controlled trial (GAIN). *Trials*. Jun 16 2021;22(1):401. doi:10.1186/s13063-021-05290-w

108. Elbaz S, Cinalioglu K, Sekhon K, et al. A Systematic Review of Telemedicine for Older Adults With Dementia During COVID-19: An Alternative to In-person Health Services? *Front Neurol*. 2021;12:761965. doi:10.3389/fneur.2021.761965

109. Hermann DM, Muck S, Nehen HG. Supporting dementia patients in hospital environments: health-related risks, needs and dedicated structures for patient care. *Eur J Neurol*. Feb 2015;22(2):239-45, e17-8. doi:10.1111/ene.12530

110. Bakker C, Verboom M, Koopmans R. Reimagining Postdiagnostic Care and Support in Young-Onset Dementia. *J Am Med Dir Assoc*. Feb 2022;23(2):261-265. doi:10.1016/j.jamda.2021.12.008

111. van Riet Paap J, Vernooij-Dassen M, Sommerbakk R, et al. Implementation of improvement strategies in palliative care: an integrative review. *Implement Sci*. Jul 26 2015;10(1):103. doi:10.1186/s13012-015-0293-2

112. Johl N, Patterson T, Pearson L. What do we know about the attitudes, experiences and needs of Black and minority ethnic carers of people with dementia in the United Kingdom? A systematic review of empirical research findings. *Dementia (London)*. Jul 2016;15(4):721-42. doi:10.1177/1471301214534424

113. Grundberg A, Sandberg J, Craftman AG. Childrens' and young adults' perspectives of having a parent with dementia diagnosis: A scoping review. *Dementia (London)*. Nov 2021;20(8):2933-2956. doi:10.1177/14713012211023653

114. Curnow E, Rush R, Maciver D, Gorska S, Forsyth K. Exploring the needs of people with dementia living at home reported by people with dementia and informal caregivers: a systematic review and Meta-analysis. *Aging Ment Health*. Mar 2021;25(3):397-407. doi:10.1080/13607863.2019.1695741

115. Garcia-Vivar C, Konradsen H, Kolbrun Svavarsdottir E, et al. Healthcare interventions for older people with dementia and family caregivers in Europe: A scoping review. *Int J Nurs Pract*. Apr 2024;30(2):e13172. doi:10.1111/ijn.13172

116. Atoyebi O, Eng JJ, Routhier F, Bird ML, Mortenson WB. A systematic review of systematic reviews of needs of family caregivers of older adults with dementia. *Eur J Ageing*. Sep 2022;19(3):381-396. doi:10.1007/s10433-021-00680-0

117. Khanassov V, Vedel I. Family Physician-Case Manager Collaboration and Needs of Patients With Dementia and Their Caregivers: A Systematic Mixed Studies Review. *Ann Fam Med*. Mar 2016;14(2):166-77. doi:10.1370/afm.1898

118. Millenaar JK, Bakker C, Koopmans RT, Verhey FR, Kurz A, de Vugt ME. The care needs and experiences with the use of services of people with young-onset dementia and their caregivers: a systematic review. *Int J Geriatr Psychiatry*. Dec 2016;31(12):1261-1276. doi:10.1002/gps.4502

119. Waligora KJ, Bahouth MN, Han HR. The Self-Care Needs and Behaviors of Dementia Informal Caregivers: A Systematic Review. *Gerontologist*. Sep 17 2019;59(5):e565-e583. doi:10.1093/geront/gny076

120. Vseteckova J, Deepak-Gopinath M, Borgstrom E, et al. Barriers and facilitators to adherence to group exercise in institutionalized older people living with dementia: a systematic review. *Eur Rev Aging Phys Act*. 2018;15(1):11. doi:10.1186/s11556-018-0200-3

121. Lillekroken D, Halvorsrud L, Gulesto R, Bjorge H. Family caregivers' experiences of providing care for family members from minority ethnic groups living with dementia: A qualitative systematic review. *J Clin Nurs*. May 2023;32(9-10):1625-1641. doi:10.1111/jocn.16127

122. Mangili S, Capolongo S. Healthcare Facilities and Dementia Development of a Framework to Assess Design Quality. *Stud Health Technol Inform*. Sep 2 2022;297:323-330. doi:10.3233/SHTI220856

123. Vseteckova J, Dadova K, Gracia R, et al. Barriers and facilitators to adherence to walking group exercise in older people living with dementia in the community: a systematic review. *Eur Rev Aging Phys Act*. 2020;17(1):15. doi:10.1186/s11556-020-00246-6

124. Duran-Kirac G, Uysal-Bozkir O, Uittenbroek R, van Hout H, Broese van Groenou MI. Accessibility of health care experienced by persons with dementia from ethnic minority groups and formal and informal caregivers: A scoping review of European literature. *Dementia (London)*. Feb 2022;21(2):677-700. doi:10.1177/14713012211055307

125. Phenwan T, Sixsmith J, McSwiggan L, Buchanan D. A narrative review of facilitating and inhibiting factors in advance care planning initiation in people with dementia. *Eur Geriatr Med*. Jun 2020;11(3):353-368. doi:10.1007/s41999-020-00314-1

126. McCabe M, You E, Tatangelo G. Hearing Their Voice: A Systematic Review of Dementia Family Caregivers' Needs. *Gerontologist*. Oct 2016;56(5):e70-88. doi:10.1093/geront/gnw078

127. Tilburgs B, Vernooij-Dassen M, Koopmans R, van Gennip H, Engels Y, Perry M. Barriers and facilitators for GPs in dementia advance care planning: A systematic integrative review. *PLoS One*. 2018;13(6):e0198535. doi:10.1371/journal.pone.0198535

128. van Alphen HJ, Hortobagyi T, van Heuvelen MJ. Barriers, motivators, and facilitators of physical activity in dementia patients: A systematic review. *Arch Gerontol Geriatr*. Sep-Oct 2016;66:109-18. doi:10.1016/j.archger.2016.05.008

129. Clare L, Kudlicka A, Oyebode JR, et al. Individual goal-oriented cognitive rehabilitation to improve everyday functioning for people with early-stage dementia: A multicentre randomised controlled trial (the GREAT trial). *Int J Geriatr Psychiatry*. May 2019;34(5):709-721. doi:10.1002/gps.5076

130. Zygouris S, Gkioka M, Moraitou D, et al. Views of nursing staff on computerized dementia screening : A validation and pilot study in a general hospital. *Z Gerontol Geriatr*. Nov 2019;52(Suppl 4):258-263. Einstellungen des Pflegepersonals zum computergestutzten Demenz-Screening : Eine Validierung und Pilotstudie in einem Allgemeinkrankenhaus. doi:10.1007/s00391-019-01633-0
