# Supplementary material for: The needs and unmet needs for people living with dementia, caregivers and care workers in dementia health care systems: a systematic review
Source: Front Public Health. 2025 Aug 20;13:1605993. doi: 10.3389/fpubh.2025.1605993 (PMC12404970; doi:10.3389/fpubh.2025.1605993)
Supplement: Supplementary file 2 [file Table_2.docx]

| **RefID** | **First author** | **YEAR** | **OVERALL**  **SCORE** | **SCREENING**  **QUESTIONS** | | **QUALITATIVE STUDIES** | | | | | **QUANTITATIVE DESCRIPTIVE STUDIES** | | | | | **MIXED-METHODS STUDIES** | | | | |
| --- | --- | --- | --- | --- | --- | --- | --- | --- | --- | --- | --- | --- | --- | --- | --- | --- | --- | --- | --- | --- |
|  |  |  |  | S1. Are there clear research questions? | S2. Do the collected data allow to address the research questions? | 1.1. Is the qualitative approach appropriate to answer the research question? | 1.2. Are the qualitative data collection methods adequate to address the research question? | 1.3. Are the findings adequately derived from the data? | 1.4. Is the interpretation of results sufficiently substantiated by data? | 1.5. Is there coherence between qualitative data sources, collection, analysis and interpretation? | 4.1. Is the sampling strategy relevant to address the research question? | 4.2. Is the sample representative of the target population? | 4.3. Are the measurements appropriate? | 4.4. Is the risk of nonresponse bias low? | 4.5. Is the statistical analysis appropriate to answer the research question? | 5.1. Is there an adequate rationale for using a mixed methods design to address the research question? | 5.2. Are the different components of the study effectively integrated to answer the research question? | 5.3. Are the outputs of the integration of qualitative and quantitative components adequately interpreted? | 5.4. Are divergences and inconsistencies between quantitative and qualitative results adequately addressed? | 5.5. Do the different components of the study adhere to the quality criteria of each tradition of the methods involved? |
| [1] | Abreu W et al. | 2019 | 100% | Yes | Yes |  |  |  |  |  | Yes | Yes | Yes | Yes | Yes |  |  |  |  |  |
| [2] | Bakker C et al. | 2014 | 100% | Yes | Yes |  |  |  |  |  | Yes | Yes | Yes | Yes | Yes |  |  |  |  |  |
| [3] | Bakker C et al. | 2013 | 100% | Yes | Yes |  |  |  |  |  | Yes | Yes | Yes | Yes | Yes |  |  |  |  |  |
| [4] | Barry HE et al. | 2021 | 100% | Yes | Yes | Yes | Yes | Yes | Yes | Yes |  |  |  |  |  |  |  |  |  |  |
| [5] | Bökberg C et al. | 2014 | 100% | Yes | Yes | Yes | Yes | Yes | Yes | Yes |  |  |  |  |  |  |  |  |  |  |
| [6] | Bökberg C et al. | 2018 | 100% | Yes | Yes |  |  |  |  |  | Yes | Yes | Yes | Yes | Yes |  |  |  |  |  |
| [7] | Carcavilla N et al. | 2021 | 80% | Yes | Yes |  |  |  |  |  | Yes | Yes | Yes | No | Yes |  |  |  |  |  |
| [8] | Claudia Miranda-Castillo et al. | 2013 | 100% | Yes | Yes |  |  |  |  |  | Yes | Yes | Yes | Yes | Yes |  |  |  |  |  |
| [9] | De Cola MC et al. | 2017 | 100% | Yes | Yes |  |  |  |  |  | Yes | Yes | Yes | Yes | Yes |  |  |  |  |  |
| [10] | Dibao-Dina C et al. | 2022 | 100% | Yes | Yes | Yes | Yes | Yes | Yes | Yes | Yes | Yes | Yes | Yes | Yes | Yes | Yes | Yes | Yes | Yes |
| [11] | Dickinson C et al. | 2013 | 100% | Yes | Yes | Yes | Yes | Yes | Yes | Yes |  |  |  |  |  |  |  |  |  |  |
| [12] | Eichler T et al. | 2016 | 100% | Yes | Yes |  |  |  |  |  | Yes | Yes | Yes | Yes | Yes |  |  |  |  |  |
| [13] | Farina N et al. | 2021 | 100% | Yes | Yes | Yes | Yes | Yes | Yes | Yes |  |  |  |  |  |  |  |  |  |  |
| [14] | Felicity Smith et al. | 2014 | 100% | Yes | Yes | Yes | Yes | Yes | Yes | Yes |  |  |  |  |  |  |  |  |  |  |
| [15] | Foley T et al. | 2020 | 100% | Yes | Yes | Yes | Yes | Yes | Yes | Yes |  |  |  |  |  |  |  |  |  |  |
| [16] | Frias CE et al. | 2020 | 80% | Yes | Yes |  |  |  |  |  | Yes | Yes | Yes | Can't tell | Yes |  |  |  |  |  |
| [17] | Froelich L et al. | 2021 | 100% | Yes | Yes |  |  |  |  |  | Yes | Yes | Yes | Yes | Yes |  |  |  |  |  |
| [18] | Giebel C et al. | 2020 | 100% | Yes | Yes | Yes | Yes | Yes | Yes | Yes |  |  |  |  |  |  |  |  |  |  |
| [19] | Gove D et al. | 2017 | 100% | Yes | Yes | Yes | Yes | Yes | Yes | Yes |  |  |  |  |  |  |  |  |  |  |
| [20] | Hossain MZ et al. | 2020 | 100% | Yes | Yes | Yes | Yes | Yes | Yes | Yes |  |  |  |  |  |  |  |  |  |  |
| [21] | Janssen N et al. | 2020 | 100% | Yes | Yes |  |  |  |  |  | Yes | Yes | Yes | Yes | Yes |  |  |  |  |  |
| [22] | Janssen N et al. | 2018 | 100% | Yes | Yes |  |  |  |  |  | Yes | Yes | Yes | Yes | Yes |  |  |  |  |  |
| [23] | Kerpershoek L et al. | 2018 | 100% | Yes | Yes |  |  |  |  |  | Yes | Yes | Yes | Yes | Yes |  |  |  |  |  |
| [24] | Leroi I et al. | 2022 | 100% | Yes | Yes | Yes | Yes | Yes | Yes | Yes | Yes | Yes | Yes | Yes | Yes | Yes | Yes | Yes | Yes | Yes |
| [25] | Malthouse R et al. | 2014 | 100% | Yes | Yes | Yes | Yes | Yes | Yes | Yes |  |  |  |  |  |  |  |  |  |  |
| [26] | Mank A et al. | 2023 | 80% | Yes | Yes |  |  |  |  |  | Yes | Yes | Yes | Can't tell | Yes |  |  |  |  |  |
| [27] | Mazurek J et al. | 2019 | 100% | Yes | Yes |  |  |  |  |  | Yes | Yes | Yes | Yes | Yes |  |  |  |  |  |
| [28] | Michelet M et al. | 2022 | 100% | Yes | Yes |  |  |  |  |  | Yes | Yes | Yes | Yes | Yes |  |  |  |  |  |
| [29] | Minaya-Freire A et al. | 2020 | 100% | Yes | Yes | Yes | Yes | Yes | Yes | Yes |  |  |  |  |  |  |  |  |  |  |
| [30] | Mitchell G et al. | 2020 | 100% | Yes | Yes | Yes | Yes | Yes | Yes | Yes |  |  |  |  |  |  |  |  |  |  |
| [31] | Monsees J et al. | 2020 | 100% | Yes | Yes | Yes | Yes | Yes | Yes | Yes |  |  |  |  |  |  |  |  |  |  |
| [32] | Moreno-Cámara S et al. | 2019 | 100% | Yes | Yes | Yes | Yes | Yes | Yes | Yes |  |  |  |  |  |  |  |  |  |  |
| [33] | Nielsen TR et al. | 2021 | 100% | Yes | Yes | Yes | Yes | Yes | Yes | Yes |  |  |  |  |  |  |  |  |  |  |
| [34] | Nienke van Wezel et al. | 2014 | 100% | Yes | Yes | Yes | Yes | Yes | Yes | Yes |  |  |  |  |  |  |  |  |  |  |
| [35] | Oliveira D et al. | 2019 | 100% | Yes | Yes | Yes | Yes | Yes | Yes | Yes |  |  |  |  |  |  |  |  |  |  |
| [36] | Page S et al. | 2013 | 80% | Yes | Yes |  |  |  |  |  | Yes | Yes | Yes | Can't tell | Yes |  |  |  |  |  |
| [37] | Pini S et al. | 2018 | 100% | Yes | Yes | Yes | Yes | Yes | Yes | Yes |  |  |  |  |  |  |  |  |  |  |
| [38] | Quinn C et al. | 2019 | 100% | Yes | Yes |  |  |  |  |  | Yes | Yes | Yes | Yes | Yes |  |  |  |  |  |
| [39] | Rusowicz J et al. | 2021 | 100% | Yes | Yes |  |  |  |  |  | Yes | Yes | Yes | Yes | Yes |  |  |  |  |  |
| [40] | Ryan L et al. | 2021 | 100% | Yes | Yes | Yes | Yes | Yes | Yes | Yes |  |  |  |  |  |  |  |  |  |  |
| [41] | Saloua Berdai Chaouni et al. | 2018 | 100% | Yes | Yes | Yes | Yes | Yes | Yes | Yes |  |  |  |  |  |  |  |  |  |  |
| [42] | Schnelli A et al. | 2021 | 100% | Yes | Yes | Yes | Yes | Yes | Yes | Yes |  |  |  |  |  |  |  |  |  |  |
| [43] | Somme D et al. | 2013 | 100% | Yes | Yes | Yes | Yes | Yes | Yes | Yes |  |  |  |  |  |  |  |  |  |  |
| [44] | Telenius EW et al. | 2020 | 100% | Yes | Yes | Yes | Yes | Yes | Yes | Yes |  |  |  |  |  |  |  |  |  |  |
| [45] | Timmons S et al. | 2021 | 80% | Yes | Yes |  |  |  |  |  | Yes | Yes | Yes | Can't tell | Yes |  |  |  |  |  |
| [46] | Varik M et al. | 2020 | 100% | Yes | Yes | Yes | Yes | Yes | Yes | Yes |  |  |  |  |  |  |  |  |  |  |
| [47] | Vullings I et al. | 2020 | 100% | Yes | Yes | Yes | Yes | Yes | Yes | Yes |  |  |  |  |  |  |  |  |  |  |

**Supplementary Table 2**. Quality of the selected papers according to the revised version of the Mixed Methods Appraisal Tool (MMAT) [48].

References

1. Abreu W, Tolson D, Jackson GA, Staines H, Costa N. The relationship between frailty, functional dependence, and healthcare needs among community-dwelling people with moderate to severe dementia. *Health Soc Care Community*. May 2019;27(3):642-653. doi:10.1111/hsc.12678

2. Bakker C, de Vugt ME, van Vliet D, et al. The relationship between unmet care needs in young-onset dementia and the course of neuropsychiatric symptoms: a two-year follow-up study. *Int Psychogeriatr*. Dec 2014;26(12):1991-2000. doi:10.1017/S1041610213001476

3. Bakker C, de Vugt ME, van Vliet D, et al. Unmet needs and health-related quality of life in young-onset dementia. *Am J Geriatr Psychiatry*. Nov 2014;22(11):1121-30. doi:10.1016/j.jagp.2013.02.006

4. Barry HE, McGrattan M, Ryan C, et al. 'I just take them because I know the people that give them to me': A theory-informed interview study of community-dwelling people with dementia and carers' perspectives of medicines management. *Int J Geriatr Psychiatry*. Jun 2021;36(6):883-891. doi:10.1002/gps.5488

5. Bokberg C, Ahlstrom G, Karlsson S, Hallberg IR, Janlov AC. Best practice and needs for improvement in the chain of care for persons with dementia in Sweden: a qualitative study based on focus group interviews. *BMC Health Serv Res*. Nov 30 2014;14(1):596. doi:10.1186/s12913-014-0596-z

6. Bokberg C, Ahlstrom G, Karlsson S. Utilisation of formal and informal care and services at home among persons with dementia: a cross-sectional study. *Scand J Caring Sci*. Jun 2018;32(2):843-851. doi:10.1111/scs.12515

7. Carcavilla N, Pozo AS, Gonzalez B, et al. Needs of Dementia Family Caregivers in Spain During the COVID-19 Pandemic. *J Alzheimers Dis*. 2021;80(2):533-537. doi:10.3233/JAD-201430

8. Miranda-Castillo C, Woods B, Orrell M. The needs of people with dementia living at home from user, caregiver and professional perspectives: a cross-sectional survey. *BMC Health Serv Res*. Feb 4 2013;13(1):43. doi:10.1186/1472-6963-13-43

9. De Cola MC, Lo Buono V, Mento A, et al. Unmet Needs for Family Caregivers of Elderly People With Dementia Living in Italy: What Do We Know So Far and What Should We Do Next? *Inquiry*. Jan 1 2017;54:46958017713708. doi:10.1177/0046958017713708

10. Dibao-Dina C, Oger C, Foley T, et al. Intermediate care in caring for dementia, the point of view of general practitioners: A key informant survey across Europe. *Front Med (Lausanne)*. 2022;9:1016462. doi:10.3389/fmed.2022.1016462

11. Dickinson C, Bamford C, Exley C, Emmett C, Hughes J, Robinson L. Planning for tomorrow whilst living for today: the views of people with dementia and their families on advance care planning. *Int Psychogeriatr*. Dec 2013;25(12):2011-21. doi:10.1017/S1041610213001531

12. Eichler T, Thyrian JR, Hertel J, et al. Unmet Needs of Community-Dwelling Primary Care Patients with Dementia in Germany: Prevalence and Correlates. *J Alzheimers Dis*. 2016;51(3):847-55. doi:10.3233/JAD-150935

13. Farina N, Williams A, Clarke K, et al. Barriers, motivators and facilitators of physical activity in people with dementia and their family carers in England: dyadic interviews. *Aging Ment Health*. Jun 2021;25(6):1115-1124. doi:10.1080/13607863.2020.1727852

14. Smith F, Grijseels MS, Ryan P, Tobiansky R. Assisting people with dementia with their medicines: experiences of family carers. *Int J Pharm Pract*. Feb 2015;23(1):44-51. doi:10.1111/ijpp.12158

15. Foley T, Sheehan C, Jennings AA, O'Sullivan T. A qualitative study of the dementia-care experiences and educational needs of physiotherapists in the Republic of Ireland. *Physiotherapy*. Jun 2020;107:267-274. doi:10.1016/j.physio.2019.08.006

16. Frias CE, Cabrera E, Zabalegui A. Informal Caregivers' Roles in Dementia: The Impact on Their Quality of Life. *Life (Basel)*. Oct 23 2020;10(11)doi:10.3390/life10110251

17. Froelich L, Llado A, Khandker RK, et al. Quality of Life and Caregiver Burden of Alzheimer's Disease Among Community Dwelling Patients in Europe: Variation by Disease Severity and Progression. *J Alzheimers Dis Rep*. 2021;5(1):791-804. doi:10.3233/ADR-210025

18. Giebel C, Cannon J, Hanna K, et al. Impact of COVID-19 related social support service closures on people with dementia and unpaid carers: a qualitative study. *Aging Ment Health*. Jul 2021;25(7):1281-1288. doi:10.1080/13607863.2020.1822292

19. Gove D, Small N, Downs M, Vernooij-Dassen M. General practitioners' perceptions of the stigma of dementia and the role of reciprocity. *Dementia (London)*. Oct 2017;16(7):948-964. doi:10.1177/1471301215625657

20. Hossain MZ, Khan HTA. Barriers to access and ways to improve dementia services for a minority ethnic group in England. *J Eval Clin Pract*. Dec 2020;26(6):1629-1637. doi:10.1111/jep.13361

21. Janssen N, Handels RL, Kohler S, et al. Profiles of Met and Unmet Needs in People with Dementia According to Caregivers' Perspective: Results from a European Multicenter Study. *J Am Med Dir Assoc*. Nov 2020;21(11):1609-1616 e1. doi:10.1016/j.jamda.2020.05.009

22. Janssen N, Handels RL, Skoldunger A, et al. Impact of Untimely Access to Formal Care on Costs and Quality of Life in Community Dwelling People with Dementia. *J Alzheimers Dis*. 2018;66(3):1165-1174. doi:10.3233/JAD-180531

23. Kerpershoek L, de Vugt M, Wolfs C, et al. Needs and quality of life of people with middle-stage dementia and their family carers from the European Actifcare study. When informal care alone may not suffice. *Aging Ment Health*. Jul 2018;22(7):897-902. doi:10.1080/13607863.2017.1390732

24. Leroi I, Wolski L, Charalambous AP, et al. Support care needs of people with hearing and vision impairment in dementia: a European cross-national perspective. *Disabil Rehabil*. Sep 2022;44(18):5069-5081. doi:10.1080/09638288.2021.1923071

25. Malthouse R, Fox F. Exploring experiences of physical activity among people with Alzheimer's disease and their spouse carers: a qualitative study. *Physiotherapy*. Jun 2014;100(2):169-75. doi:10.1016/j.physio.2013.10.002

26. Mank A, van Maurik IS, Rijnhart JJM, et al. Determinants of informal care time, distress, depression, and quality of life in care partners along the trajectory of Alzheimer's disease. *Alzheimers Dement (Amst)*. Apr-Jun 2023;15(2):e12418. doi:10.1002/dad2.12418

27. Mazurek J, Szczesniak D, Urbanska K, Droes RM, Rymaszewska J. Met and unmet care needs of older people with dementia living at home: Personal and informal carers' perspectives. *Dementia (London)*. Aug 2019;18(6):1963-1975. doi:10.1177/1471301217733233

28. Michelet M, Selbaek G, Strand BH, et al. Associations between unmet needs for daytime activities and company and scores on the Neuropsychiatric Inventory-Questionnaire in people with dementia: a longitudinal study. *Aging Ment Health*. Apr 2022;26(4):725-734. doi:10.1080/13607863.2021.1910792

29. Minaya-Freire A, Ramon-Aribau A, Pou-Pujol G, Fajula-Bonet M, Subirana-Casacuberta M. Facilitators, Barriers, and Solutions in Pain Management for Older Adults with Dementia. *Pain Manag Nurs*. Dec 2020;21(6):495-501. doi:10.1016/j.pmn.2020.03.003

30. Mitchell G, McTurk V, Carter G, Brown-Wilson C. Emphasise capability, not disability: exploring public perceptions, facilitators and barriers to living well with dementia in Northern Ireland. *BMC Geriatr*. Dec 3 2020;20(1):525. doi:10.1186/s12877-020-01933-w

31. Monsees J, Schmachtenberg T, Hoffmann W, et al. Dementia in People with a Turkish Migration Background: Experiences and Utilization of Healthcare Services. *J Alzheimers Dis*. 2020;77(2):865-875. doi:10.3233/JAD-200184

32. Moreno-Camara S, Palomino-Moral PA, Moral-Fernandez L, Frias-Osuna A, Parra-Anguita L, Del-Pino-Casado R. Perceived Needs of The Family Caregivers of People with Dementia in a Mediterranean Setting: A Qualitative Study. *Int J Environ Res Public Health*. Mar 19 2019;16(6)doi:10.3390/ijerph16060993

33. Nielsen TR, Nielsen DS, Waldemar G. Barriers in access to dementia care in minority ethnic groups in Denmark: a qualitative study. *Aging Ment Health*. Aug 2021;25(8):1424-1432. doi:10.1080/13607863.2020.1787336

34. van Wezel N, Francke AL, Kayan-Acun E, Ljm Deville W, van Grondelle NJ, Blom MM. Family care for immigrants with dementia: The perspectives of female family carers living in The Netherlands. *Dementia (London)*. Jan 2016;15(1):69-84. doi:10.1177/1471301213517703

35. Oliveira D, Zarit SH, Orrell M. Health-Promoting Self-Care in Family Caregivers of People With Dementia: The Views of Multiple Stakeholders. *Gerontologist*. Sep 17 2019;59(5):e501-e511. doi:10.1093/geront/gnz029

36. Page S, Hope K. Towards new ways of working in dementia: perceptions of specialist dementia care nurses about their own level of knowledge, competence and unmet educational needs. *J Psychiatr Ment Health Nurs*. Aug 2013;20(6):549-56. doi:10.1111/jpm.12029

37. Pini S, Ingleson E, Megson M, Clare L, Wright P, Oyebode JR. A Needs-led Framework for Understanding the Impact of Caring for a Family Member With Dementia. *Gerontologist*. Mar 19 2018;58(2):e68-e77. doi:10.1093/geront/gnx148

38. Quinn C, Nelis SM, Martyr A, et al. Influence of Positive and Negative Dimensions of Dementia Caregiving on Caregiver Well-Being and Satisfaction With Life: Findings From the IDEAL Study. *Am J Geriatr Psychiatry*. Aug 2019;27(8):838-848. doi:10.1016/j.jagp.2019.02.005

39. Rusowicz J, Pezdek K, Szczepanska-Gieracha J. Needs of Alzheimer's Charges' Caregivers in Poland in the Covid-19 Pandemic-An Observational Study. *Int J Environ Res Public Health*. Apr 23 2021;18(9)doi:10.3390/ijerph18094493

40. Ryan L. Accessing community dementia care services in Ireland: Emotional barriers for caregivers. *Health Soc Care Community*. Nov 2021;29(6):1980-1989. doi:10.1111/hsc.13342

41. Berdai Chaouni S, De Donder L. Invisible realities: Caring for older Moroccan migrants with dementia in Belgium. *Dementia (London)*. Oct-Nov 2019;18(7-8):3113-3129. doi:10.1177/1471301218768923

42. Schnelli A, Hirt J, Zeller A. Persons with dementia as internet users: what are their needs? A qualitative study. *J Clin Nurs*. Mar 2021;30(5-6):849-860. doi:10.1111/jocn.15629

43. Somme D, Gautier A, Pin S, Corvol A. General practitioner's clinical practices, difficulties and educational needs to manage Alzheimer's disease in France: analysis of national telephone-inquiry data. *BMC Fam Pract*. Jun 13 2013;14(1):81. doi:10.1186/1471-2296-14-81

44. Telenius EW, Eriksen S, Rokstad AMM. I need to be who I am: a qualitative interview study exploring the needs of people with dementia in Norway. *BMJ Open*. Aug 16 2020;10(8):e035886. doi:10.1136/bmjopen-2019-035886

45. Timmons S, O'Loughlin C, Buckley C, et al. Dementia palliative care: A multi-site survey of long term care STAFF'S education needs and readiness to change. *Nurse Educ Pract*. Mar 2021;52:103006. doi:10.1016/j.nepr.2021.103006

46. Varik M, Medar M, Saks K. Informal caregivers' experiences of caring for persons with dementia in Estonia: A narrative study. *Health Soc Care Community*. Mar 2020;28(2):448-455. doi:10.1111/hsc.12877

47. Vullings I, Labrie N, Wammes JD, de Bekker-Grob EW, MacNeil-Vroomen J. Important components for Dutch in-home care based on qualitative interviews with persons with dementia and informal caregivers. *Health Expect*. Dec 2020;23(6):1412-1419. doi:10.1111/hex.13118

48. Hong QN, Fàbregues S, Bartlett G, et al. The Mixed Methods Appraisal Tool (MMAT) version 2018 for information professionals and researchers. *Education for Information*. 2018;34(4):285-291. doi:10.3233/efi-180221
